# Integrative Molecular Analyses of an Individual Transcription Factor-Based Genomic Model for Lung Cancer Prognosis

**DOI:** 10.1155/2021/5125643

**Published:** 2021-12-07

**Authors:** Rong Yao, Leilei Zhou, Zhongying Guo, Dahong Zhang, Tiecheng Zhang

**Affiliations:** ^1^Department of Medical Oncology, The Affiliated Huaian No.1 People's Hospital of Nanjing Medical University, Huai'an, 223300 Jiangsu, China; ^2^Department of Pathology, The Affiliated Huaian No.1 People's Hospital of Nanjing Medical University, Huai'an, 223300 Jiangsu, China

## Abstract

**Objective:**

Precision medicine with molecular profiles has revolutionized the management of lung cancer contributing to improved prognosis. Herein, we aimed to uncover the gene expression profiling of transcription factors (TFs) in lung cancer as well as to develop a TF-based genomic model.

**Methods:**

We retrospectively curated lung cancer patients from public databases. Through comparing mRNA expression profiling between lung cancer and normal specimens, specific TFs were determined. Thereafter, a TF genomic model was developed with univariate Cox regression and stepwise multivariable Cox analyses, which was verified through the GSE72094 dataset. Gene set enrichment analyses (GSEA) were presented. Downstream targets of TFs were predicted with ChEA, JASPAR, and MotifMap projects, and their biological significance was investigated through the clusterProfiler algorithm.

**Results:**

In the TCGA cohort, we proposed a TF-based genomic model, comprised of SATB2, HLF, and NPAS2. Lung cancer individuals were remarkably stratified into high- and low-risk groups. Survival analyses uncovered that high-risk populations presented unfavorable survival outcomes. ROCs confirmed the excellent predictive potency in patients' prognosis. Additionally, this model was an independent prognostic indicator in accordance with multivariate analyses. The clinical implication of the model was well verified in an independent dataset. High risk score was in relation to carcinogenic pathways. Downstream targets were characterized by immune and carcinogenic activation.

**Conclusion:**

The proposed TF genomic model acts as a promising marker for estimation of lung cancer patients' outcomes. Prospective research is required for testing the clinical utility of the model in individualized management of lung cancer.

## 1. Introduction

Lung cancer occupies the major cause of cancer incidence and mortality across the globe, with estimated 2.1 million newly diagnosed cases as well as 18.4% of all cancer-related deaths in 2018 [1]. It is primarily classified into small cell lung cancer (SCLC) as well as non-small cell lung cancer (NSCLC). Among them, NSCLC occupies around 85% of lung cancer cases, with an undesirable five-year survival rate below 16%. It is comprised of lung adenocarcinoma (LUAD) and lung squamous cell carcinoma (LUSC), as well as large cell carcinoma histological subtypes [2]. Chemotherapy represents the major therapeutic option against lung cancer, and platinum-based dual treatment is the standard treatment against advanced patients [3]. Nevertheless, chemotherapeutic resistance remains challenging, impeding much therapeutic success. Though tyrosine kinase-based targeted therapy interferes with the oncogenic pathway in NSCLC, acquired resistance evolves contributing to rapid disease recurrence and progression [4]. Immunotherapy like PD-1 and PD-L1 inhibitors has displayed superiority to traditional chemotherapy, which has become the standard for treating NSCLC [5–7]. Nevertheless, in past clinical trials, the objective response rate of immunotherapy remains low [8].

Transcription factors (TFs), DNA-binding proteins, recognize the promoter sequences of genes as well as subsequently guide gene expression [9]. Recent studies demonstrate that patterns of TF programs as well as immune pathway activation characterize four main SCLC subtypes with diverse treatment vulnerabilities [10]. Additionally, system-epigenomics inference of TF activities indicates inactivated aryl-hydrocarbon-receptor as an important event during lung carcinogenesis [11]. Experimental evidences suggest the lung carcinogenic roles of TFs. For instance, Oct4, a critical stemness TF, controls expression of lncRNAs NEAT1 and MALAT1 for promoting lung carcinogenesis [12]. TF NFIB is in relation to tumor aggressiveness in LUAD [13]. TF OCT1 triggers the Warburg effect through upregulating hexokinase 2 in NSCLC [14]. TF YY1 mediates lung cancer progression through activation of lncRNA-PVT1 [15]. Hence, characterization of the genomic profiling of TFs may assist in offering an in-depth understanding of the precise treatment of lung cancer. Herein, we developed a TF genomic model for lung cancer outcomes and uncovered the relevant signaling pathways.

## 2. Materials and Methods

### 2.1. Public mRNA Expression Cohorts

The Cancer Genome Atlas- (TCGA-) LUAD and TCGA-LUSC datasets containing mRNA expression profiling, survival and clinicopathologic data (*n* = 522) were curated from the Genomic Data Commons tool (https://portal.gdc.cancer.gov/) [16], as a training set. Fragments per kilobase million (FPKM) were converted to transcripts per million (TPM). Gene expression arrays of 442 LUAD patients were harvested from GSE72094 [17] via the Gene Expression Omnibus (GEO) repository (https://www.ncbi.nlm.nih.gov/gds/), which acted as a testing set. This dataset was based on the GPL15048 platform. Probe IDs were transformed into gene symbols in accordance with platform annotation file. The standardized expression value was logarithmically converted as well as scaled. Thereafter, the mean expression of genes with various probes was utilized as their expression value. The corresponding patient's clinical data in TCGA and GSE72094 cohorts are displayed in Supplementary table 1. The gene sets of TFs were harvested from published literature (Supplementary table 2) [9].

### 2.2. Analyses of Lung Cancer-Specific TFs

DESeq2 package [18] was adopted for differential analyses of sequencing data. TFs with ∣log2 fold change (FC) | >1 and adjusted *p* < 0.05 were set as thresholds of lung cancer-specific TFs. Volcano plots and heat map were conducted for visualizing the distribution of lung cancer-specific TFs between lung cancer and normal specimens.

### 2.3. Construction of a TF Genomic Model

Univariate Cox regression models were conducted for evaluation of the interactions of lung cancer-specific TFs with patients' prognosis in the TCGA cohort. Thereafter, stepwise multivariate Cox regression analyses were utilized for shrinking the variables as well as screening the most predictive biomarkers. The candidate TF-specific TFs were utilized for creating a risk scoring formula that was determined through a linear integration of mRNA expression as well as matched regression coefficients derived from the stepwise multivariate Cox analyses. Through ranking of the risk scoring system, patients were stratified into high- and low-risk subpopulations. Kaplan–Meier methods were presented for estimating the survival outcomes between high- and low-risk subpopulations, and log-rank tests were adopted for the calculation of the discrepancy in prognosis between subpopulations. The receiver operator characteristic (ROC) curve was depicted through the pROC package [19]. The prognostic potency of the TF genomic model was externally verified in the GSE72094 cohort.

### 2.4. Uni- and Multivariate Cox Regression Analyses

Through univariate Cox regression analyses, the interactions of clinicopathological indicators (age, gender, and pathological staging) and TF genomic model with lung cancer prognosis were evaluated across lung cancer individuals. Thereafter, multivariate Cox regression models were established for uncovering the independent prognostic indicators.

### 2.5. Gene Set Enrichment Analyses (GESA)

Through the Java program (http://software.broadinstitute.org/gsea/index.jsp), GSEA [20] was carried out on the basis of the “c2.cp.kegg.v7.2.symbols” gene set curated from the Molecular Signatures Database (MSigDB) project [21]. Cytoscape software [22] was adopted for visualizing our GSEA results. The interactions of specific gene sets with risk score for all genes were investigated, and positively and negatively correlated ones to the enrichment score were calculated. In total, 1000 permutations were conducted, and pathways with false discovery rate (FDR) < 0.05 were regarded as having prominent enrichment.

### 2.6. Prediction of Downstream Targets of TFs

Three web-based interactive applications, containing ChIP Enrichment Analysis (ChEA; http://amp.pharm.mssm.edu/lib/chea.jsp) [23], JASPAR (http://jaspar.genereg.net), and MotifMap (http://motifmap.igb.uci.edu/) databases, were adopted for estimating the downstream targets of TFs. The ChEA project offers data on eukaryotic TFs, consensus binding sequences, and experimentally validated binding sites as well as modulated genes [24]. The JASPAR project represents an open-access project of curated, nonredundant TF-binding profiling stored as a position frequency matrix for TFs among diverse species in six taxonomic populations [25]. MotifMap provides integrative genome-wide maps of regulated motif sites for model species [26].

### 2.7. Functional Enrichment Analyses

Gene Ontology (GO) as well as Kyoto Encyclopedia of Genes and Genomes (KEGG) pathway enrichment analyses were carried out for determining the biological functions of downstream targets of TFs through the clusterProfiler package [27]. GO depicted three complementary biological concepts containing biological process (BP) and molecular function (MF) as well as cellular component (CC). Meanwhile, KEGG may assist uncover high-level functions and utilities of biological systems.

### 2.8. Statistical Analyses

All statistical analyses were managed through R software (version 3.6.3). Student's *t*-test or Wilcoxon test was adopted for statistical comparisons, with *p* < 0.05 as statistical significance.

## 3. Results

### 3.1. Identification of Lung Cancer-Specific TFs

**Figure 1:**
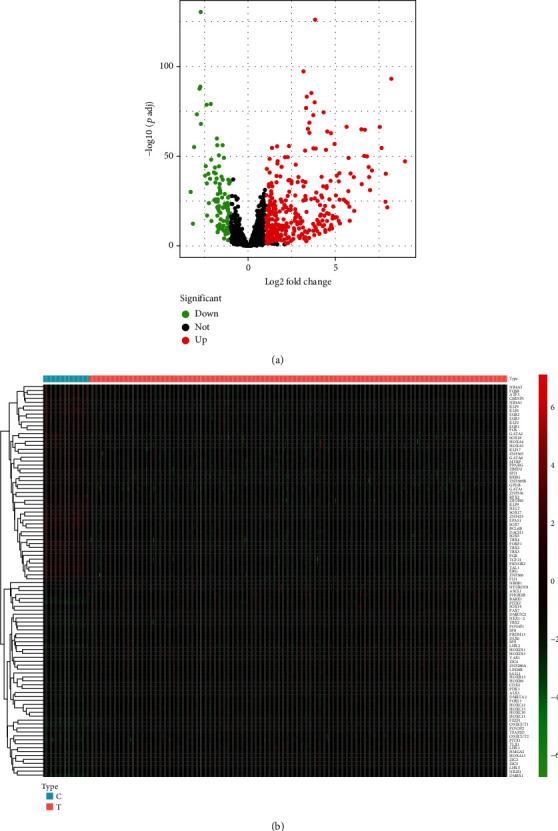
Identification of lung cancer-specific TFs utilizing mRNA expression profiling from TCGA cohort. (a) Volcano plots display lung cancer-specific TFs through comparison of differential TFs between lung cancer and normal specimens in accordance with ∣log2 FC | >1 and adjusted *p* < 0.05 thresholds. Red dots are indicative of upregulated TFs, while green dots are indicative of downregulated TFs. *x*-axis represents log2 FC while *y*-axis represents -log10 (adjusted *p*). (b) Hierarchical clustering analyses show the deregulated expression patterns of lung cancer-specific TFs in lung cancer (T) and normal (N) specimens. *x*-axis represents each sample while *y*-axis represents the RNA expression of TFs.

This study retrospectively curated mRNA expression profiling, survival, and clinicopathologic data of lung cancer from the TCGA project. With the ∣log2 FC | >1 and adjusted *p* < 0.05 thresholds, 320 upregulated TFs as well as 103 downregulated TFs were determined in lung cancer in comparison with normal tissues (Figures 1(a) and 1(b); Supplementary table 3). The above deregulated TFs were regarded as lung cancer-specific TFs.

### 3.2. Determination of Prognostic Lung Cancer-Specific TFs

**Figure 2:**
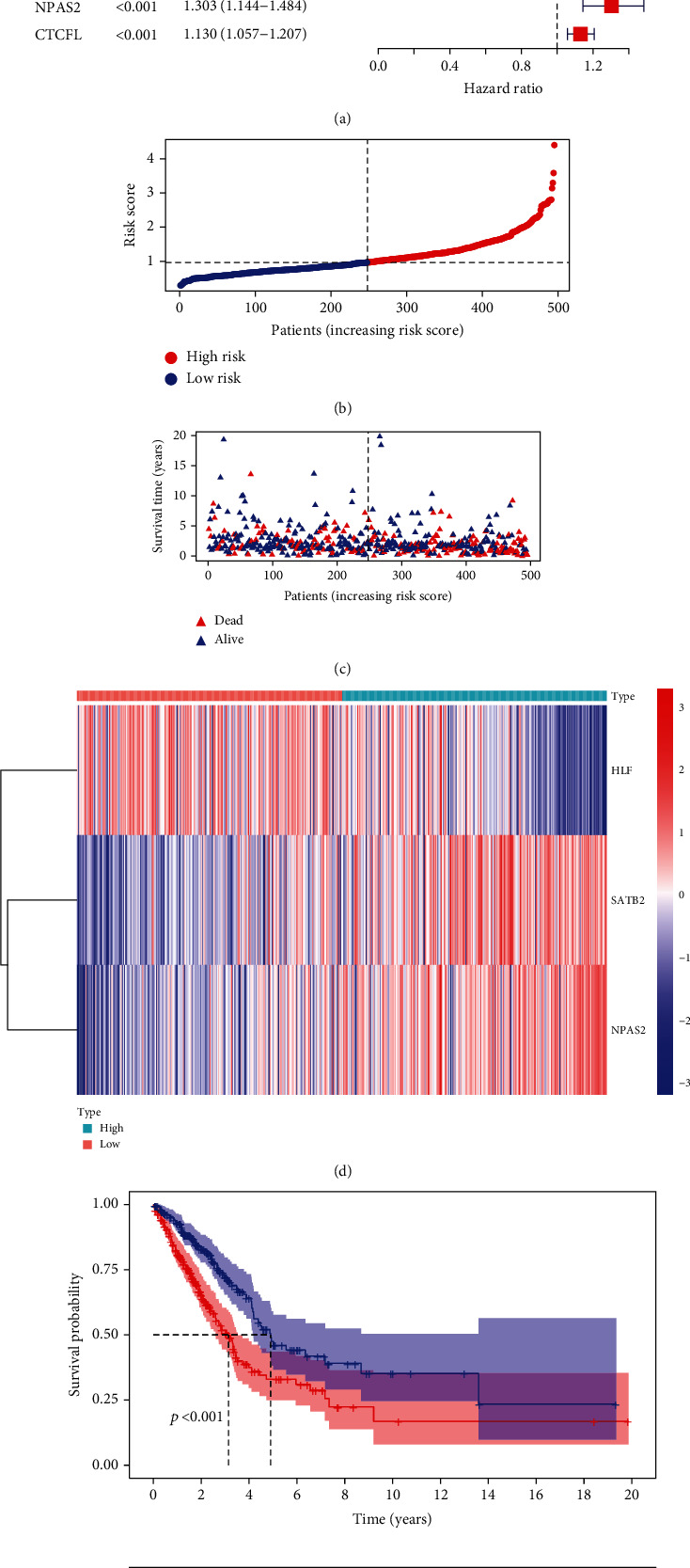
Development of a TF genomic model for lung cancer prognosis in TCGA cohort. (a) Forest diagram displays the prognostic lung cancer-specific TFs through univariate Cox regression analyses. Vertical dashed line indicates HR = 1. Green represents HR < 1 while red represents HR > 1. (b) Risk score of lung cancer patients is quantified in line with the regression coefficient derived from multivariate Cox regression models and expression value of SATB2, HLF, and NPAS2. Vertical dashed line expresses the grouping cutoff. Red dots represent high-risk specimens, while blue dots represent low-risk specimens. (c) Scatter diagram displays the survival duration and status of lung cancer patients that are ranked by risk score. Vertical dashed line expresses the grouping cutoff. Red triangle is indicative of high-risk specimen, while blue triangle is indicative of low-risk specimen. (d) Hierarchical clustering analyses present the expression patterns of SATB2, HLF, and NPAS2 in two groups. Red indicates high expression, and blue indicates low expression. (e) Survival analyses are conducted between high- and low-risk groups, and survival difference is estimated with log-rank test. (f) ROC curves are conducted on the basis of risk score for lung cancer outcomes.

On the basis of mRNA expression profiling and survival information of lung cancer patients from the TCGA cohort, we conducted the interactions of lung cancer-specific TFs with clinical prognosis through univariate Cox regression models. In accordance with *p* < 0.05, 13 lung cancer-specific TFs displayed remarkable associations with lung cancer outcomes (Figure 2(a); Table 1). Among them, GFI1B, HLF, and ZNF750 acted as protective factors of lung cancer prognosis. Meanwhile, CTCFL, TFAP2A, CENPA, VAX1, E2F7, FOXM1, SATB2, ARNTL2, NPAS2, and HMGA1 acted as risk factors of lung cancer prognosis.

### 3.3. Development of a TF Genomic Model for Lung Cancer Prognosis

Further multivariate Cox regression analyses displayed that SATB2, HLF, and NPAS2 were independently predictive of lung cancer prognosis. In accordance with the regression coefficient derived from multivariate Cox regression models and expression value of SATB2, HLF, and NPAS2, a TF genomic model was conducted for lung cancer prognosis. The risk score of each lung cancer patient in the TCGA cohort was calculated in line with the following formula: risk score = 0.215678015946362∗SATB2 expression + (−0.133926273065041)∗HLF expression + 0.22354783465585∗NPAS2 expression. Thereafter, lung cancer patients in the TCGA cohort were separated into two groups following the median risk score. Patients with >median risk score were classified into the high-risk group, while those with <median risk score were classified into the low-risk group (Figure 2(b)). Additionally, Figure 2(c) displayed the discrepancy in survival status between two groups. With the increase in risk score, the number of dead patients was gradually elevated. In comparison to the low-risk group, HLF presented reduced expression while SATB2 and NPAS2 possessed upregulated expression in the high-risk group (Figure 2(d)). Survival analyses uncovered that the high-risk group displayed remarkable survival outcomes in comparison to the low-risk group (Figure 2(e)). Further ROC analyses were conducted for verifying the predictive potency of the TF genomic model in lung cancer outcomes. The area under the curve (AUC) was 0.676, indicative of the convincing predictive potency (Figure 2(f)).

### 3.4. External Verification of the TF Genomic Model

**Figure 3:**
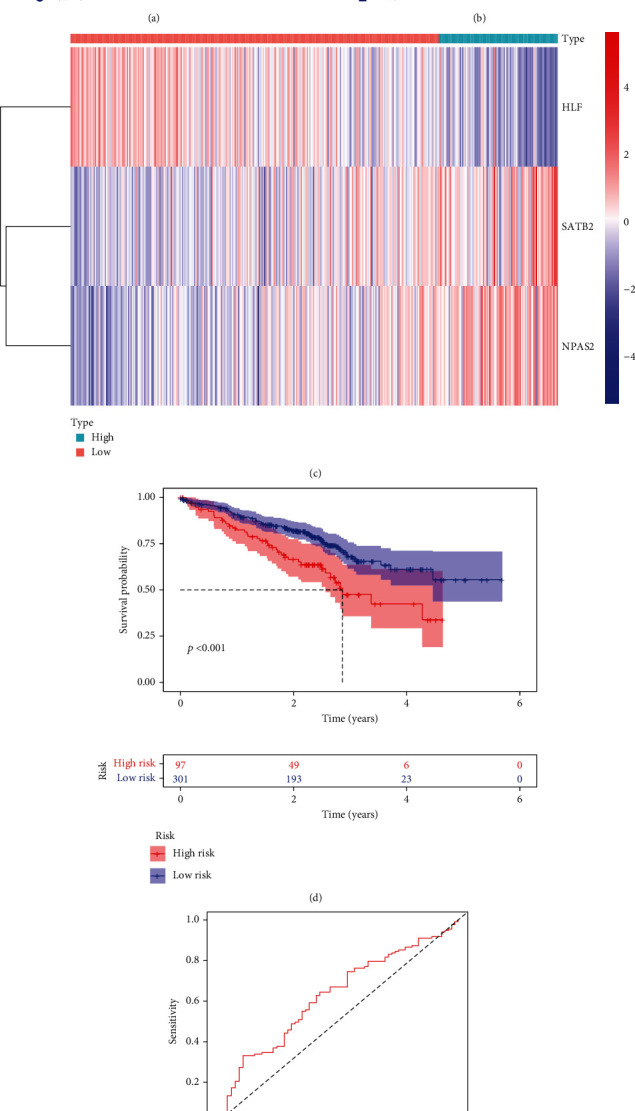
External verification of the TF genomic model for lung cancer prognosis in GSE72094 cohort. (a) Risk score distribution of lung cancer patients is displayed in line with the regression coefficient derived from multivariate Cox regression models and expression value of SATB2, HLF, and NPAS2. Vertical dashed line shows the grouping cutoff. Red dots are indicative of high-risk specimens, while blue dots are indicative of low-risk specimens. (b) Scatter plots present the survival duration and status of lung cancer patients that are ranked by risk score. Vertical dashed line represents the grouping cutoff. Red triangle expresses high-risk individual, while blue triangle expresses low-risk individual. (c) Hierarchical clustering analyses show the expression patterns of SATB2, HLF, and NPAS2 in two groups. Red represents upregulated expression, while blue represents downregulated expression. (d) Survival analyses are carried out between groups, and survival difference is estimated through log-rank test. (e) ROC curves display the predictive potency of risk score for lung cancer outcomes.

The clinical application potential of the TF genomic model was externally verified in the GSE72094 cohort. In accordance with the same formula, the risk score of each lung cancer patient was quantified. Thereafter, we clustered patients into high- and low-risk groups (Figure 3(a)). As expected, more dead patients were noted in the high-risk group (Figure 3(b)). There was enhanced expression of HLF as well as weakened expression of SATB2 and NPAS2 in the low-risk group (Figure 3(c)). Additionally, the high risk score presented more undesirable survival outcomes (Figure 3(d)). ROC curves confirmed the favorable predictive potency of the TF genomic model in lung cancer outcomes (AUC = 0.619; Figure 3(e)).

### 3.5. Analyses and Verification of the TF Genomic Model as an Independent Prognostic Indicator of Lung Cancer

**Figure 4:**
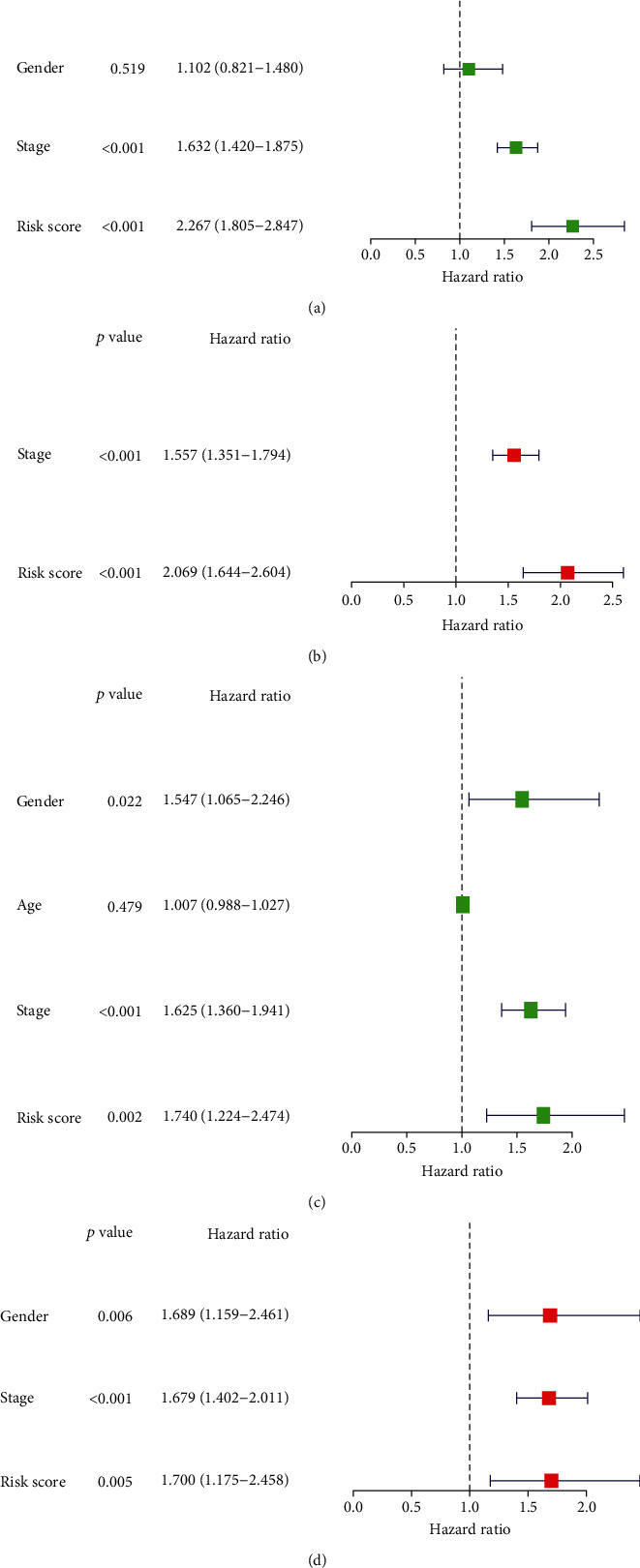
Analyses and verification of the TF genomic model as an independent prognostic indicator of lung cancer. (a) Forest diagram depicts the interactions of age, gender, and staging as well as risk score with lung cancer outcomes through univariate Cox regression models in TCGA cohort. (b) Forest diagram presents the independency of stage and risk score in prediction of lung cancer prognosis in TCGA cohort. (c) Forest diagram shows the associations of age, gender, staging, and risk score with lung cancer prognosis in GSE72094 cohort. (d) Forest diagram displays the independency of gender and staging as well as risk score in predicting patients' outcomes in GSE72094 cohort.

In the TCGA cohort, uni- and multivariate Cox regression models were presented for investigation of the interaction of conventional clinicopathological indicators and risk score with lung cancer outcomes. As a result, staging as well as risk score was independently predictive of patients' prognosis (Figures 4(a) and 4(b)). The predictive independency was externally verified in the GSE72094 cohort. Our data confirmed that gender and staging as well as risk score were independent risk factors of lung cancer outcomes (Figures 4(c) and 4(d)).

### 3.6. Analyses of the TF Genomic Model Associated with Activation of Signaling Pathways

**Figure 5:**
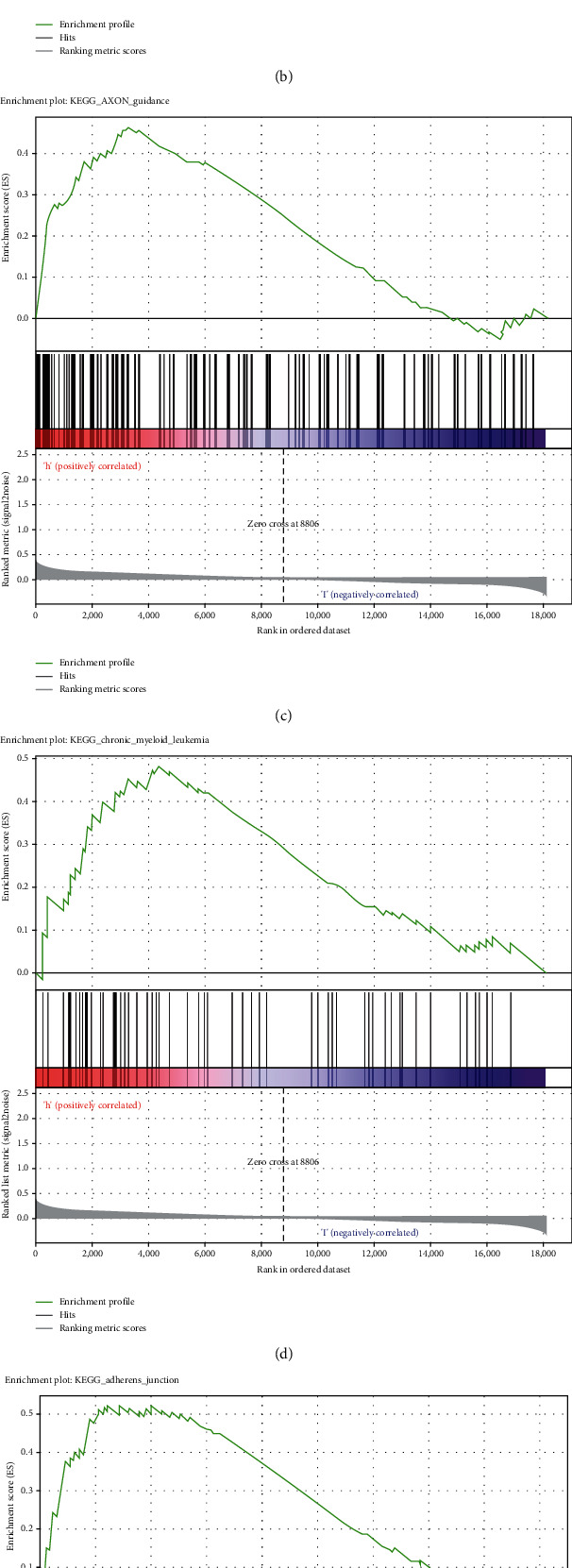
Analyses of the TF genomic model associated with activation of signaling pathways. (a–f) GSEA results present activated KEGG signaling pathways in high-risk specimens, containing (a) ECM receptor interaction, (b) small cell lung cancer, (c) axon guidance, (d) chronic myeloid leukemia, (e) adherens junction, and (f) regulation of actin cytoskeleton.

**Figure 6:**
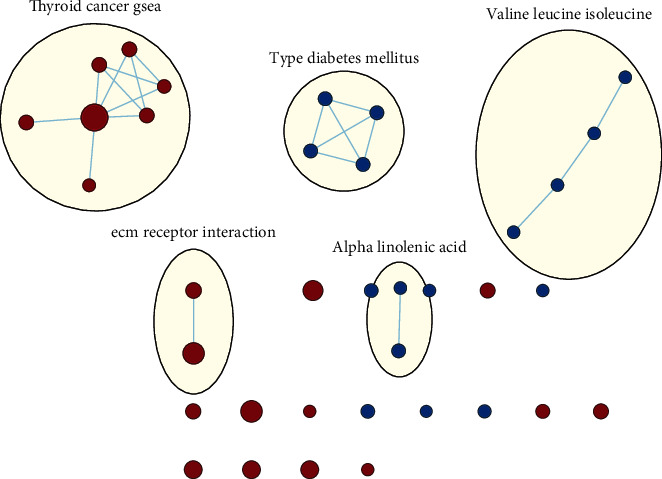
GSEA of KEGG pathways correlated to the TF genomic model. Enriched gene set is expressed through each node that is grouped in accordance with the similarity for creating a network. The size of each node displays the positive association with the number of genes as well as the thickness of line-interacting nodes indicating the proportions of common genes between nodes.

GSEA was conducted for investigating the activated signaling pathways correlated to the TF genomic model. In accordance with FDR < 0.05, ECM receptor interaction, small cell lung cancer, axon guidance, chronic myeloid leukemia, and adherens junction as well as regulation of actin cytoskeleton were remarkably activated in high-risk specimens (Figures 5(a)–5(f)). Additionally, clusters of relevant genes linked to the high risk score were determined, containing genes relating to thyroid cancer, type diabetes mellitus, valine leucine isoleucine, and ECM receptor interaction as well as alpha linolenic acid (Figure 6).

### 3.7. Determination of Downstream Targets of TFs: SATB2, HLF, and NPAS2

**Figure 7:**
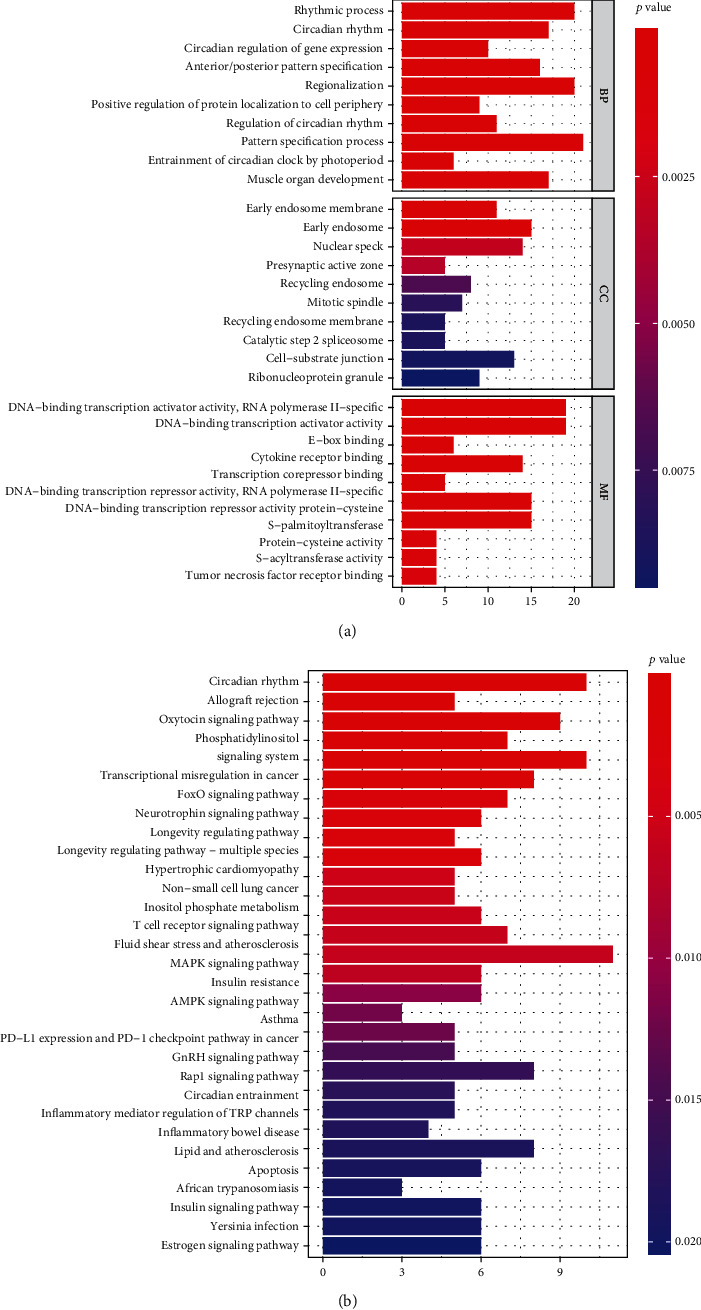
Functional enrichment analyses of downstream targets of TFs: SATB2, HLF, and NPAS2. (a) GO enrichment results enriched by downstream targets of three TFs. (b) KEGG pathway enrichment results enriched by downstream targets of three TFs.

Through integration analyses of the ChEA, JASPAR, and MotifMap databases, we determined 307 downstream targets of HLF, 4 downstream targets (CRY2, PER1, PER2, and CRY1) of NPAS2, and 2 downstream targets (UPF3B and TP63) of SATB2 (Supplementary table 4). GO enrichment analyses uncovered that the rhythmic process was remarkably enriched by downstream targets (Figure 7(a); Table 2). Additionally, we noted that the above downstream targets presented remarkable interactions with circadian rhythm and immune activation pathways (like allograft rejection, T cell receptor signaling pathway, PD-L1 expression and PD-1 checkpoint pathway in cancer, inflammatory mediator regulation of TRP channels, and inflammatory bowel disease) as well as carcinogenic pathways (like transcriptional misregulation in cancer, FoxO signaling pathway, non-small cell lung cancer, MAPK signaling pathway, AMPK signaling pathway, Rap1 signaling pathway, and apoptosis; Figure 7(b) and Table 3).

## 4. Discussion

High-throughput sequencing technologies may assist in determining more biomarkers that present close interactions with patients' outcomes at the genetic levels. Herein, we proposed a 3-TF genomic model linked to lung cancer progression through conducting reliable bioinformatic analyses. Additionally, the 3-TF genomic model acted as an independent molecular marker for prediction of lung cancer patients' survival outcomes. Our findings might be of great significance to elucidate the underlying biological mechanisms of lung carcinogenesis as well as to develop innovative prognostic indicators and molecular therapeutic targets.

Previously, a 7-TF gene model has been established for prediction of colon adenocarcinoma outcomes [28]. A 9-TF signature can be predictive of breast cancer recurrence for optimizing clinical management [29]. Additionally, Yang et al. proposed a TF-based prognostic signature that reliably predicts endometrial cancer individuals' survival outcomes [30]. Here, through univariate analyses followed by stepwise multivariate Cox regression analyses, we developed a TF genomic model for lung cancer outcomes. In accordance with the formula, a TF genomic model was conducted, containing SATB2, HLF, and NPAS2. ROC curves confirmed the reliability of this model in prediction of patients' prognosis. Following integration of clinicopathological indicators, the model was independently predictive of clinical prognosis. To our knowledge, the 3-TF genomic model's potential as a predictor has not been proposed in previous evidences, though research might offer a novel guide of lung cancer outcomes. In routine clinical practice, pathological staging acts as an important survival determinant concerning oncologists as well as lung cancer individuals. Nevertheless, diverse patients' survival outcomes with the same staging are indicative of the deficient pathological staging system for prognosis on the basis of the anatomic scope and staging system of the disease, in which pathological changes reflect the biological heterogeneity within lung cancer. The issues influence the predictive potency of the conventional system for lung cancer individuals. Our GSEA uncovered that the 7-TF genomic model presented remarkable interactions with ECM receptor interaction, SCLC, axon guidance, chronic myeloid leukemia, and adherens junction as well as regulation of actin cytoskeleton, indicative of the interactions of the 7-TF genomic model with lung carcinogenesis.

Previous evidences suggest the biological significance of SATB2, HLF, and NPAS2 within the TF-based genomic model in lung carcinogenesis. For instance, SATB2 reduces NSCLC invasiveness through modulation of EMT-relevant proteins as well as histone methylation of G9a [31]. Further, hypoxic tumor-derived exosomal miR-31-5p triggers LUAD metastases through negative modulation of SATB2-reversed EMT as well as activation of the MEK/ERK pathway [32]. Downregulated HLF facilitates multiple-organ distant metastases of NSCLC via the PPAR/NF-*κ*b pathway NSCLC [33]. Reduced HLF expression is predictive of undesirable clinical outcomes of LUAD [34]. NPAS2 polymorphism independently predicts NSCLC patients' prognosis [35]. Further analyses determined the downstream targets of TFs: SATB2, HLF, and NPAS2. Our further biological function analyses demonstrated the interactions of these downstream targets with circadian rhythm and immune activation pathways (like allograft rejection, T cell receptor signaling pathway, PD-L1 expression and PD-1 checkpoint pathway in cancer, inflammatory mediator regulation of TRP channels, and inflammatory bowel disease) and carcinogenic pathways (like transcriptional misregulation in cancer, FoxO signaling pathway, NSCLC, MAPK signaling pathway, AMPK signaling pathway, Rap1 signaling pathway, and apoptosis), indicating that SATB2, HLF, and NPAS2 modulated the above pathways to participate in lung carcinogenesis.

Nevertheless, a few limitations of this study need to be pointed out. Our findings were primarily on the basis of integrated bioinformatic analyses. However, sufficient experimental verification of our results remains lacking. In future studies, we will conduct in-depth in vitro and in vivo experiments to verify our conclusion. Because all patients were retrospectively harvested, the underlying bias linked to unbalanced clinicopathological characteristics cannot be ignored. Additionally, the reliability of the 3-TF genomic model for predicting survival outcomes of lung cancer individuals remains a key issue in the clinic. In particular, the guideline for the clinical application of our 3-TF genomic model requires an in-depth definition in our future studies.

## 5. Conclusion

Collectively, our findings proposed and verified a 3-TF genomic model (SATB2, HLF, and NPAS2) for prediction of lung cancer outcomes. This genomic model acted as an independent indicator as well as a complement prognostic factor for clinicopathological features of lung cancer.

## Figures and Tables

**Table 1:** Univariate Cox regression models identify prognostic lung cancer-specific TFs.

TFs	HR	Lower 95% CI	Upper 95% CI	*P* value
ARNTL2	1.302281	1.169475	1.450168	1.49*E*-06
VAX1	1.210274	1.131859	1.294122	2.35*E*-08
CENPA	1.19974	1.084774	1.326889	0.000395
SATB2	1.278489	1.112124	1.469741	0.000552
GFI1B	0.776427	0.683637	0.881812	9.75*E*-05
FOXM1	1.272198	1.139285	1.420617	1.90*E*-05
E2F7	1.263852	1.135898	1.406221	1.71*E*-05
HLF	0.86293	0.801083	0.929552	0.000102
TFAP2A	1.188719	1.092255	1.293702	6.24*E*-05
ZNF750	0.881095	0.821778	0.944694	0.000371
HMGA1	1.356436	1.184181	1.553747	1.08*E*-05
NPAS2	1.302838	1.143688	1.484135	6.90*E*-05
CTCFL	1.129607	1.056855	1.207367	0.000333

**Table 2:** GO enrichment results enriched by downstream targets of three TFs.

Description	Gene ratio	BgRatio	*p* value	FDR	*q* value	Count
Rhythmic process	20/277	294/18862	1.48*E*-08	3.08*E*-05	2.45*E*-05	20
Circadian rhythm	17/277	212/18862	1.67*E*-08	3.08*E*-05	2.45*E*-05	17
Circadian regulation of gene expression	10/277	67/18862	4.71*E*-08	5.21*E*-05	4.14*E*-05	10
Anterior/posterior pattern specification	16/277	203/18862	5.64*E*-08	5.21*E*-05	4.14*E*-05	16
Regionalization	20/277	326/18862	8.22*E*-08	6.07*E*-05	4.83*E*-05	20
Positive regulation of protein localization to cell periphery	9/277	67/18862	5.67*E*-07	0.000349	0.000277	9
Regulation of circadian rhythm	11/277	118/18862	1.34*E*-06	0.000665	0.000529	11
Pattern specification process	21/277	426/18862	1.44*E*-06	0.000665	0.000529	21
Entrainment of circadian clock by photoperiod	6/277	29/18862	3.40*E*-06	0.001394	0.001108	6
Muscle organ development	17/277	317/18862	4.90*E*-06	0.001716	0.001365	17
Early endosome membrane	11/283	162/19520	2.53*E*-05	0.008847	0.008168	11
Early endosome	15/283	378/19520	0.00044	0.076941	0.07104	15
Nuclear speck	14/283	411/19520	0.002834	0.255165	0.235596	14
Presynaptic active zone	5/283	70/19520	0.003472	0.255165	0.235596	5
Recycling endosome	8/283	190/19520	0.006657	0.255165	0.235596	8
Mitotic spindle	7/283	157/19520	0.008067	0.255165	0.235596	7
Recycling endosome membrane	5/283	87/19520	0.008695	0.255165	0.235596	5
Catalytic step 2 spliceosome	5/283	87/19520	0.008695	0.255165	0.235596	5
Cell-substrate junction	13/283	423/19520	0.009108	0.255165	0.235596	13
Ribonucleoprotein granule	9/283	244/19520	0.009482	0.255165	0.235596	9
DNA-binding transcription activator activity, RNA polymerase II-specific	19/285	443/18337	6.93*E*-05	0.008115	0.007448	19
DNA-binding transcription activator activity	19/285	447/18337	7.80*E*-05	0.008115	0.007448	19
E-box binding	6/285	47/18337	8.41*E*-05	0.008115	0.007448	6
Cytokine receptor binding	14/285	270/18337	8.94*E*-05	0.008115	0.007448	14
Transcription corepressor binding	5/285	30/18337	9.07*E*-05	0.008115	0.007448	5
DNA-binding transcription repressor activity, RNA polymerase II-specific	15/285	307/18337	9.81*E*-05	0.008115	0.007448	15
DNA-binding transcription repressor activity	15/285	309/18337	0.000105	0.008115	0.007448	15
Protein-cysteine S-palmitoyltransferase activity	4/285	28/18337	0.000872	0.052206	0.047919	4
Protein-cysteine S-acyltransferase activity	4/285	28/18337	0.000872	0.052206	0.047919	4
Tumor necrosis factor receptor binding	4/285	31/18337	0.001292	0.069613	0.063896	4

**Table 3:** KEGG pathway enrichment results enriched by downstream targets of three TFs.

Description	Gene ratio	BgRatio	*p* value	FDR	*q* value	Count
Circadian rhythm	10/126	31/8101	1.94*E*-11	4.71*E*-09	3.93*E*-09	10
Allograft rejection	5/126	38/8101	0.00028	0.033838	0.028259	5
Oxytocin signaling pathway	9/126	154/8101	0.000636	0.038062	0.031787	9
Phosphatidylinositol signaling system	7/126	97/8101	0.000751	0.038062	0.031787	7
Transcriptional misregulation in cancer	10/126	192/8101	0.000786	0.038062	0.031787	10
FoxO signaling pathway	8/126	131/8101	0.000959	0.038685	0.032308	8
Neurotrophin signaling pathway	7/126	119/8101	0.002472	0.065225	0.054472	7
Longevity regulating pathway	6/126	89/8101	0.002547	0.065225	0.054472	6
Longevity regulating pathway—multiple species	5/126	62/8101	0.002679	0.065225	0.054472	5
Hypertrophic cardiomyopathy	6/126	90/8101	0.002695	0.065225	0.054472	6
Non-small cell lung cancer	5/126	72/8101	0.005121	0.095997	0.080171	5
Inositol phosphate metabolism	5/126	73/8101	0.005431	0.095997	0.080171	5
T cell receptor signaling pathway	6/126	104/8101	0.00551	0.095997	0.080171	6
Fluid shear stress and atherosclerosis	7/126	139/8101	0.005836	0.095997	0.080171	7
MAPK signaling pathway	11/126	294/8101	0.00595	0.095997	0.080171	11
Insulin resistance	6/126	108/8101	0.006608	0.099953	0.083476	6
AMPK signaling pathway	6/126	120/8101	0.010857	0.154556	0.129077	6
Asthma	3/126	31/8101	0.012022	0.157127	0.131224	3
PD-L1 expression and PD-1 checkpoint pathway in cancer	5/126	89/8101	0.012336	0.157127	0.131224	5
GnRH signaling pathway	5/126	93/8101	0.01471	0.164644	0.137502	5
Rap1 signaling pathway	8/126	210/8101	0.016473	0.164644	0.137502	8
Circadian entrainment	5/126	97/8101	0.017373	0.164644	0.137502	5
Inflammatory mediator regulation of TRP channels	5/126	98/8101	0.018086	0.164644	0.137502	5
Inflammatory bowel disease	4/126	65/8101	0.018212	0.164644	0.137502	4
Lipid and atherosclerosis	8/126	215/8101	0.018715	0.164644	0.137502	8
Apoptosis	6/126	136/8101	0.019138	0.164644	0.137502	6
African trypanosomiasis	3/126	37/8101	0.019429	0.164644	0.137502	3
Insulin signaling pathway	6/126	137/8101	0.019767	0.164644	0.137502	6
Yersinia infection	6/126	137/8101	0.019767	0.164644	0.137502	6
Estrogen signaling pathway	6/126	138/8101	0.02041	0.164644	0.137502	6

## Data Availability

The data used to support the findings of this study are included within the supplementary information files.

## Abbreviations

SCLC: Small cell lung cancer
NSCLC: Non-small cell lung cancer
LUAD: Lung adenocarcinoma
LUSC: Lung squamous cell carcinoma
TFs: Transcription factors
TCGA: The Cancer Genome Atlas
FPKM: Fragments per kilobase million
TPM: Transcripts per million
GEO: Gene Expression Omnibus
FC: Fold-change
ROC: Receiver operator characteristic
GESA: Gene set enrichment analyses
MSigDB: Molecular Signatures Database
FDR: False discovery rate
ChEA: ChIP enrichment analysis
GO: Gene Ontology
KEGG: Kyoto Encyclopedia of Genes and Genomes
BP: Biological process
MF: Molecular function
CC: Cellular component.

## Supplementary Materials

Supplementary 1 Supplementary Table 1: clinical features of lung cancer patients in TCGA cohort.

Supplementary 2 Supplementary Table 2: the gene sets of TFs.

Supplementary 3 Supplementary Table 3: the detailed information of lung cancer-specific TFs.

Supplementary 4 Supplementary Table 4: determination of downstream targets of TFs: SATB2, HLF, and NPAS2.
