# Supplementary material for: Integrative Molecular Analyses of an Individual Transcription Factor-Based Genomic Model for Lung Cancer Prognosis
Source: Dis Markers. 2021 Dec 7;2021:5125643. doi: 10.1155/2021/5125643 (PMC8672105; doi:10.1155/2021/5125643)
Supplement: Supplementary 1 — Supplementary Table 1: clinical features of lung cancer patients in TCGA cohort. [file 5125643.f1.pdf]

Supplementary table 1. Clinical features of lung cancer patients in TCGA cohort.

ID	futime	fustat	age	gender	grade
TCGA-J2-8192	739	0	65	FEMALE	unknow
TCGA-91-8499	36	0	76	FEMALE	unknow
TCGA-53-7624	1043	1	40	FEMALE	unknow
TCGA-55-6986	3261	0	74	FEMALE	unknow
TCGA-38-4632	1357	1	42	MALE	unknow
TCGA-78-7146	173	1	71	FEMALE	unknow
TCGA-NJ-A4YG	2261	0	65	MALE	unknow
TCGA-86-8279	949	0	46	MALE	unknow
TCGA-50-6591	119	1	63	FEMALE	unknow
TCGA-50-5939	460	1	85	MALE	unknow
TCGA-62-8402	1498	1	73	FEMALE	unknow
TCGA-49-4506	999	1	68	FEMALE	unknow
TCGA-91-6847	842	0	62	FEMALE	unknow
TCGA-97-8547	657	0	78	FEMALE	unknow
TCGA-97-7546	1285	0	76	FEMALE	unknow
TCGA-73-4659	711	1	66	MALE	unknow
TCGA-55-A48Y	630	0	69	MALE	unknow
TCGA-50-5049	3094	0	70	MALE	unknow
TCGA-44-3396	1130	0	74	FEMALE	unknow
TCGA-05-4405	610	0	74	FEMALE	unknow
TCGA-78-8662	3361	1	53	FEMALE	unknow
TCGA-50-8460	829	0	74	MALE	unknow
TCGA-55-8087	462	0	59	FEMALE	unknow
TCGA-MP-A4TA	950	1	75	FEMALE	unknow
TCGA-55-7815	773	0	76	MALE	unknow
TCGA-55-8096	719	1	67	FEMALE	unknow
TCGA-NJ-A55R	603	0	67	MALE	unknow
TCGA-91-7771	492	0	62	MALE	unknow
TCGA-69-7760	202	0	73	MALE	unknow
TCGA-44-5644	863	0	51	FEMALE	unknow
TCGA-69-7973	230	0	42	FEMALE	unknow
TCGA-44-6145	595	0	62	FEMALE	unknow
TCGA-64-1676	1728	0	58	MALE	unknow
TCGA-97-7553	1870	0	58	FEMALE	unknow
TCGA-55-8511	552	0	73	FEMALE	unknow
TCGA-MP-A4TF	336	1	58	FEMALE	unknow
TCGA-55-8204	515	0	87	FEMALE	unknow
TCGA-55-8616	48	0	58	FEMALE	unknow
TCGA-86-8281	0	0	75	MALE	unknow
TCGA-55-7574	995	1	64	FEMALE	unknow
TCGA-L9-A743	664	0	56	MALE	unknow
TCGA-73-4670	131	0	69	FEMALE	unknow
TCGA-50-5066	1442	0	72	MALE	unknow
TCGA-75-5126		0	unknow	FEMALE	unknow
TCGA-64-5779	864	0	61	MALE	unknow
TCGA-55-6543	435	0	60	FEMALE	unknow
TCGA-55-A48Z	651	0	60	FEMALE	unknow
TCGA-86-8278	944	0	63	FEMALE	unknow
TCGA-L4-A4E5	578	0	48	FEMALE	unknow
TCGA-49-4505	428	1	61	FEMALE	unknow
TCGA-69-7980	411	0	70	FEMALE	unknow

TCGA-55-7725	442	0	68	FEMALE	unknow
TCGA-78-7162	3169	1	75	MALE	unknow
TCGA-80-5608	2832	0	unknow	FEMALE	unknow
TCGA-78-7159	1974	0	60	FEMALE	unknow
TCGA-MP-A4TI	429	1	72	MALE	unknow
TCGA-78-7542	321	1	56	MALE	unknow
TCGA-91-6836	417	0	52	FEMALE	unknow
TCGA-78-7537	1622	1	72	MALE	unknow
TCGA-97-7941	484	0	72	FEMALE	unknow
TCGA-MP-A4TJ	339	1	62	FEMALE	unknow
TCGA-78-8660	321	1	69	MALE	unknow
TCGA-49-AARQ	6732	0	41	FEMALE	unknow
TCGA-05-4415	91	1	57	MALE	unknow
TCGA-86-A4JF	737	1	56	MALE	unknow
TCGA-86-7701	947	0	66	MALE	unknow
TCGA-44-3918	1036	0	60	FEMALE	unknow
TCGA-50-6597	1268	1	79	FEMALE	unknow
TCGA-NJ-A4YI	4	1	87	FEMALE	unknow
TCGA-86-8280	701	0	54	FEMALE	unknow
TCGA-44-2666	97	1	43	MALE	unknow
TCGA-78-7154	593	1	72	MALE	unknow
TCGA-55-A491	626	0	81	FEMALE	unknow
TCGA-05-4390	1126	0	58	FEMALE	unknow
TCGA-05-4382	607	0	68	MALE	unknow
TCGA-55-8206	888	0	56	MALE	unknow
TCGA-55-7576	670	0	54	MALE	unknow
TCGA-55-8506	11	0	62	FEMALE	unknow
TCGA-50-5930	282	1	47	MALE	unknow
TCGA-05-4403	578	0	76	MALE	unknow
TCGA-55-7911	537	0	70	FEMALE	unknow
TCGA-78-7143	4961	1	62	FEMALE	unknow
TCGA-62-A46P	594	1	65	MALE	unknow
TCGA-95-8494	84	0	67	MALE	unknow
TCGA-35-5375	264	0	61	MALE	unknow
TCGA-78-7163	7248	0	60	MALE	unknow
TCGA-05-4417	455	0	51	FEMALE	unknow
TCGA-91-6828	323	0	70	MALE	unknow
TCGA-44-A47A	466	0	78	FEMALE	unknow
TCGA-L9-A443	193	1	63	FEMALE	unknow
TCGA-86-A4D0	116	1	48	MALE	unknow
TCGA-44-A479	486	0	73	FEMALE	unknow
TCGA-97-A4LX	614	0	81	MALE	unknow
TCGA-49-4486	2318	1	72	MALE	unknow
TCGA-50-5932	1235	1	75	MALE	unknow
TCGA-86-8073	740	0	58	MALE	unknow
TCGA-NJ-A55O	13	0	56	FEMALE	unknow
TCGA-55-A494	481	0	61	FEMALE	unknow
TCGA-69-7765	165	0	56	MALE	unknow
TCGA-86-8672	19	1	59	MALE	unknow
TCGA-49-4488	869	1	74	FEMALE	unknow
TCGA-55-7903	567	0	64	MALE	unknow
TCGA-MP-A4T2	1136	1	71	MALE	unknow
TCGA-49-4487	855	1	72	FEMALE	unknow

TCGA-99-8033	656	1	74	FEMALE	unknow
TCGA-50-5068	1499	1	59	FEMALE	unknow
TCGA-55-6978	176	1	81	MALE	unknow
TCGA-91-A4BC	44	0	59	MALE	unknow
TCGA-49-4490	385	1	45	FEMALE	unknow
TCGA-86-7955	1072	0	62	MALE	unknow
TCGA-05-4430	761	0	59	FEMALE	unknow
TCGA-44-3398	1163	0	77	FEMALE	unknow
TCGA-49-4507	268	1	73	FEMALE	unknow
TCGA-97-A4M5	634	0	83	MALE	unknow
TCGA-49-6745	522	0	82	MALE	unknow
TCGA-86-8674	806	0	50	MALE	unknow
TCGA-05-4384	426	0	66	MALE	unknow
TCGA-69-7979	408	0	71	FEMALE	unknow
TCGA-78-7147	586	1	67	FEMALE	unknow
TCGA-93-7347	683	0	76	FEMALE	unknow
TCGA-49-4514	1700	0	79	FEMALE	unknow
TCGA-49-6742	488	1	70	MALE	unknow
TCGA-MN-A4N1	827	0	60	MALE	unknow
TCGA-J2-A4AD	550	1	61	FEMALE	unknow
TCGA-78-7160	697	1	61	MALE	unknow
TCGA-05-4397	731	1	65	MALE	unknow
TCGA-91-8497	434	1	75	FEMALE	unknow
TCGA-55-7283	609	0	76	FEMALE	unknow
TCGA-05-4244	0	0	70	MALE	unknow
TCGA-91-6848	224	0	59	MALE	unknow
TCGA-MP-A4TD	307	1	71	MALE	unknow
TCGA-55-8620	375	1	60	MALE	unknow
TCGA-97-7547	1965	0	67	FEMALE	unknow
TCGA-44-6777	987	1	85	FEMALE	unknow
TCGA-38-4629	864	1	68	MALE	unknow
TCGA-55-8097	476	0	60	FEMALE	unknow
TCGA-97-8179	435	0	72	MALE	unknow
TCGA-78-8648	1209	1	58	FEMALE	unknow
TCGA-75-5125	2027	1	unknow	MALE	unknow
TCGA-95-7944	377	0	71	MALE	unknow
TCGA-44-6147	845	0	67	FEMALE	unknow
TCGA-55-7727	119	0	70	MALE	unknow
TCGA-95-7039	1272	0	54	FEMALE	unknow
TCGA-55-6975	118	1	61	MALE	unknow
TCGA-05-4433	730	0	82	MALE	unknow
TCGA-05-4427	791	0	65	FEMALE	unknow
TCGA-86-7711	1046	1	70	MALE	unknow
TCGA-69-7761	186	0	84	MALE	unknow
TCGA-44-2661	1159	0	69	FEMALE	unknow
TCGA-55-6981	1379	1	53	FEMALE	unknow
TCGA-MP-A4TH	741	0	70	FEMALE	unknow
TCGA-55-8299	469	1	61	FEMALE	unknow
TCGA-L4-A4E6	435	0	67	MALE	unknow
TCGA-44-A47F	337	0	74	MALE	unknow
TCGA-55-7913	561	1	61	FEMALE	unknow
TCGA-38-4628	1492	1	65	FEMALE	unknow
TCGA-67-6215	174	0	52	FEMALE	unknow

TCGA-NJ-A7XG	617	0	49	MALE	unknow
TCGA-67-3772	573	0	82	FEMALE	unknow
TCGA-S2-AA1A	513	0	68	FEMALE	unknow
TCGA-49-6767	677	0	46	FEMALE	unknow
TCGA-55-6980	2109	0	56	MALE	unknow
TCGA-44-6144	723	0	58	MALE	unknow
TCGA-44-5645	852	0	61	FEMALE	unknow
TCGA-MP-A4T7	167	1	75	FEMALE	unknow
TCGA-62-A46V	2199	0	78	FEMALE	unknow
TCGA-44-6774	658	0	56	FEMALE	unknow
TCGA-55-8621	515	0	75	FEMALE	unknow
TCGA-05-4396	303	1	76	MALE	unknow
TCGA-55-7573	487	0	72	FEMALE	unknow
TCGA-49-AARR	4992	0	68	MALE	unknow
TCGA-97-A4M3	540	0	69	FEMALE	unknow
TCGA-49-AARO	3759	0	39	FEMALE	unknow
TCGA-91-6830	60	0	65	FEMALE	unknow
TCGA-38-6178	448	0	70	FEMALE	unknow
TCGA-55-8091	600	0	74	MALE	unknow
TCGA-55-8507	418	0	53	MALE	unknow
TCGA-75-7030		0	unknow	MALE	unknow
TCGA-86-A456	896	0	78	FEMALE	unknow
TCGA-55-A492	596	0	70	FEMALE	unknow
TCGA-64-1678	1189	0	70	FEMALE	unknow
TCGA-62-8399	2696	0	62	MALE	unknow
TCGA-55-6985	1233	0	58	FEMALE	unknow
TCGA-44-8120	260	0	58	MALE	unknow
TCGA-50-5935	653	1	86	FEMALE	unknow
TCGA-50-5044	624	1	72	FEMALE	unknow
TCGA-55-8085	904	0	64	MALE	unknow
TCGA-L9-A444	307	0	60	FEMALE	unknow
TCGA-55-8615	446	0	67	MALE	unknow
TCGA-97-A4M0	652	0	60	FEMALE	unknow
TCGA-95-7562	87	1	71	MALE	unknow
TCGA-38-4626	3674	0	57	FEMALE	unknow
TCGA-67-6216	141	0	57	FEMALE	unknow
TCGA-75-6203		0	unknow	FEMALE	unknow
TCGA-67-3773	427	0	84	FEMALE	unknow
TCGA-78-7161	291	1	69	FEMALE	unknow
TCGA-NJ-A4YQ	1432	0	69	FEMALE	unknow
TCGA-50-5072	250	1	74	MALE	unknow
TCGA-50-5931	434	1	75	FEMALE	unknow
TCGA-49-AARN	1135	1	56	FEMALE	unknow
TCGA-64-1681	1167	1	61	FEMALE	unknow
TCGA-99-7458	747	0	74	FEMALE	unknow
TCGA-35-4122	225	0	69	MALE	unknow
TCGA-55-7728	704	0	64	FEMALE	unknow
TCGA-44-7672	719	0	52	FEMALE	unknow
TCGA-44-7662	218	0	61	MALE	unknow
TCGA-86-7714	625	1	61	FEMALE	unknow
TCGA-97-7552	1932	0	70	MALE	unknow
TCGA-44-5643	1013	0	53	MALE	unknow
TCGA-75-7025	3305	0	unknow	MALE	unknow

TCGA-78-8655	2360	0	77	FEMALE	unknow
TCGA-97-7937	564	0	65	MALE	unknow
TCGA-64-1679	2488	0	58	FEMALE	unknow
TCGA-86-A4P8	805	0	59	FEMALE	unknow
TCGA-55-8205	599	0	76	FEMALE	unknow
TCGA-44-2668	761	1	51	MALE	unknow
TCGA-NJ-A4YP	50	0	52	MALE	unknow
TCGA-49-4494	1081	1	77	MALE	unknow
TCGA-49-AAR9	260	1	61	MALE	unknow
TCGA-38-4625	2973	0	66	FEMALE	unknow
TCGA-44-7661	557	1	69	FEMALE	unknow
TCGA-55-7726	652	0	72	FEMALE	unknow
TCGA-75-6205		1	unknow	FEMALE	unknow
TCGA-55-6972	1632	1	72	MALE	unknow
TCGA-86-8359	444	1	52	MALE	unknow
TCGA-50-6673	22	1	84	FEMALE	unknow
TCGA-44-3917	1183	0	33	FEMALE	unknow
TCGA-95-7567	568	0	61	MALE	unknow
TCGA-97-8176	468	1	63	MALE	unknow
TCGA-64-1677	628	1	77	FEMALE	unknow
TCGA-50-5942	1847	0	67	FEMALE	unknow
TCGA-38-4627	1147	1	64	FEMALE	unknow
TCGA-86-8055	124	1	79	MALE	unknow
TCGA-55-A4DF	440	1	88	MALE	unknow
TCGA-44-2664	1251	0	66	FEMALE	unknow
TCGA-44-A47B	287	0	79	MALE	unknow
TCGA-62-A46R	1725	1	54	FEMALE	unknow
TCGA-71-8520	210	1	60	FEMALE	unknow
TCGA-80-5611	2595	0	unknow	MALE	unknow
TCGA-05-4250	121	1	79	FEMALE	unknow
TCGA-78-7158	179	1	59	FEMALE	unknow
TCGA-53-7813	424	0	51	FEMALE	unknow
TCGA-55-6970	464	1	67	FEMALE	unknow
TCGA-73-A9RS	340	1	41	MALE	unknow
TCGA-69-7764	414	0	75	MALE	unknow
TCGA-55-8094	541	0	51	MALE	unknow
TCGA-95-A4VN	553	0	62	FEMALE	unknow
TCGA-69-8254	409	0	85	MALE	unknow
TCGA-49-AAR3	1893	0	69	MALE	unknow
TCGA-49-4510	896	1	51	FEMALE	unknow
TCGA-55-7281	872	0	70	FEMALE	unknow
TCGA-69-7763	690	0	69	MALE	unknow
TCGA-78-7148	626	1	71	MALE	unknow
TCGA-97-A4M7	629	0	74	MALE	unknow
TCGA-86-8668	423	0	61	FEMALE	unknow
TCGA-49-AAQV	677	1	63	FEMALE	unknow
TCGA-71-6725	256	0	48	FEMALE	unknow
TCGA-55-7570	824	0	60	MALE	unknow
TCGA-05-4402	244	1	57	FEMALE	unknow
TCGA-67-4679	448	0	69	MALE	unknow
TCGA-05-4422	365	0	68	MALE	unknow
TCGA-55-7227	952	1	77	MALE	unknow
TCGA-55-1594	1178	0	68	MALE	unknow

TCGA-49-AAR4	879	1	51	MALE	unknow
TCGA-62-A46Y	414	1	70	FEMALE	unknow
TCGA-86-8054	1148	0	61	MALE	unknow
TCGA-62-A46S	1653	1	73	MALE	unknow
TCGA-97-8177	499	0	59	FEMALE	unknow
TCGA-05-5425	882	0	68	MALE	unknow
TCGA-49-6744	1683	0	64	FEMALE	unknow
TCGA-44-6778	1864	0	59	MALE	unknow
TCGA-75-6206	2590	0	unknow	MALE	unknow
TCGA-80-5607		0	unknow	FEMALE	unknow
TCGA-NJ-A4YF	2161	0	50	FEMALE	unknow
TCGA-78-7220	807	1	53	FEMALE	unknow
TCGA-50-6595	189	1	74	FEMALE	unknow
TCGA-78-7167	2681	1	77	MALE	unknow
TCGA-95-7948	476	0	42	FEMALE	unknow
TCGA-75-6207		1	unknow	MALE	unknow
TCGA-05-4389	1369	0	70	MALE	unknow
TCGA-05-5429	275	1	60	MALE	unknow
TCGA-55-7907	343	1	77	MALE	unknow
TCGA-67-3776	61	0	57	FEMALE	unknow
TCGA-73-4677	38	1	74	MALE	unknow
TCGA-97-8172	545	0	75	FEMALE	unknow
TCGA-75-5147	1333	0	unknow	FEMALE	unknow
TCGA-50-5045	2174	1	57	FEMALE	unknow
TCGA-44-A4SU	409	1	67	FEMALE	unknow
TCGA-55-8301	534	0	58	MALE	unknow
TCGA-55-1596	2065	0	55	MALE	unknow
TCGA-55-A4DG	608	0	71	MALE	unknow
TCGA-49-AARE	1229	1	51	FEMALE	unknow
TCGA-50-6592	777	1	71	FEMALE	unknow
TCGA-J2-A4AG	988	0	66	FEMALE	unknow
TCGA-67-3771	610	0	77	FEMALE	unknow
TCGA-73-4662	2515	0	65	FEMALE	unknow
TCGA-05-5420	457	0	67	MALE	unknow
TCGA-86-8056	139	0	63	FEMALE	unknow
TCGA-J2-A4AE	1079	0	77	FEMALE	unknow
TCGA-44-7669	574	1	59	MALE	unknow
TCGA-44-8119	285	0	73	MALE	unknow
TCGA-50-5941	1474	0	55	FEMALE	unknow
TCGA-64-5781	1559	0	55	FEMALE	unknow
TCGA-73-4666	800	0	52	FEMALE	unknow
TCGA-L9-A8F4	476	0	64	FEMALE	unknow
TCGA-69-8253	426	0	59	FEMALE	unknow
TCGA-97-8552	626	0	55	FEMALE	unknow
TCGA-44-6776	2616	0	60	FEMALE	unknow
TCGA-L9-A5IP	58	1	40	FEMALE	unknow
TCGA-44-6148	704	0	60	MALE	unknow
TCGA-38-4630	1073	1	75	FEMALE	unknow
TCGA-95-7947	477	0	67	MALE	unknow
TCGA-4B-A93V	300	1	52	FEMALE	unknow
TCGA-86-8076	993	0	42	MALE	unknow
TCGA-05-4410	0	0	62	MALE	unknow
TCGA-62-A472	910	0	70	MALE	unknow

TCGA-55-6968	1293	1	61	MALE	unknow
TCGA-55-8514	520	0	70	FEMALE	unknow
TCGA-75-7031		0	unknow	FEMALE	unknow
TCGA-62-8397	1289	0	70	FEMALE	unknow
TCGA-86-7953	997	0	69	FEMALE	unknow
TCGA-75-5146	2368	0	unknow	MALE	unknow
TCGA-44-A47G	351	0	73	FEMALE	unknow
TCGA-86-8671	839	0	72	FEMALE	unknow
TCGA-86-8358	653	0	44	MALE	unknow
TCGA-93-A4JN	718	0	71	MALE	unknow
TCGA-05-4432	761	0	66	MALE	unknow
TCGA-86-8669	938	0	64	MALE	unknow
TCGA-95-A4VK	651	0	74	FEMALE	unknow
TCGA-44-7670	882	0	47	FEMALE	unknow
TCGA-73-7499	1531	1	81	FEMALE	unknow
TCGA-62-A46U	2067	0	71	FEMALE	unknow
TCGA-55-8508	617	0	60	FEMALE	unknow
TCGA-44-2657	1351	0	74	FEMALE	unknow
TCGA-97-8171	568	0	81	MALE	unknow
TCGA-50-5944	1750	0	69	FEMALE	unknow
TCGA-78-7633	1528	1	67	MALE	unknow
TCGA-64-5775	62	1	71	MALE	unknow
TCGA-J2-8194	724	0	69	FEMALE	unknow
TCGA-78-7539	791	0	75	FEMALE	unknow
TCGA-91-6829	1258	1	78	MALE	unknow
TCGA-99-8028	1118	0	50	FEMALE	unknow
TCGA-78-7153	3635	0	65	FEMALE	unknow
TCGA-L9-A50W	442	1	75	MALE	unknow
TCGA-50-6594	370	1	79	FEMALE	unknow
TCGA-50-5936	257	1	58	MALE	unknow
TCGA-05-5428	670	0	57	MALE	unknow
TCGA-55-5899	930	0	58	MALE	unknow
TCGA-44-7659	691	0	70	MALE	unknow
TCGA-55-7816	468	1	49	FEMALE	unknow
TCGA-05-4425	669	0	70	FEMALE	unknow
TCGA-44-2665	1301	0	55	FEMALE	unknow
TCGA-05-4424	913	0	70	MALE	unknow
TCGA-78-7156	976	1	62	MALE	unknow
TCGA-49-4512	905	1	69	FEMALE	unknow
TCGA-78-7536	244	1	69	MALE	unknow
TCGA-97-8175	551	0	55	FEMALE	unknow
TCGA-49-4501	1421	1	67	FEMALE	unknow
TCGA-53-7626	929	1	76	FEMALE	unknow
TCGA-55-6984	760	1	71	FEMALE	unknow
TCGA-44-2659	1367	0	65	FEMALE	unknow
TCGA-MP-A4SW	1778	1	53	MALE	unknow
TCGA-55-A48X	689	0	63	FEMALE	unknow
TCGA-55-1592	701	1	65	MALE	unknow
TCGA-86-8673	862	0	61	MALE	unknow
TCGA-35-4123	182	0	38	MALE	unknow
TCGA-78-8640	7062	0	59	MALE	unknow
TCGA-75-7027	3059	0	unknow	MALE	unknow
TCGA-99-8032	44	0	61	MALE	unknow

TCGA-49-6761	354	0	68	FEMALE	unknow
TCGA-MP-A4SY	1501	1	61	MALE	unknow
TCGA-55-A490	99	1	78	MALE	unknow
TCGA-93-7348	531	0	75	FEMALE	unknow
TCGA-49-AAR0	4765	0	57	MALE	unknow
TCGA-93-A4JP	578	0	64	MALE	unknow
TCGA-55-6979	237	1	59	FEMALE	unknow
TCGA-73-7498	1189	0	58	FEMALE	unknow
TCGA-MP-A4T8	161	1	68	MALE	unknow
TCGA-50-6590	1288	1	72	FEMALE	unknow
TCGA-64-5815	866	0	74	MALE	unknow
TCGA-78-7540	1197	1	66	FEMALE	unknow
TCGA-55-8510	539	0	55	FEMALE	unknow
TCGA-44-7671	889	0	64	MALE	unknow
TCGA-91-8496	505	0	63	FEMALE	unknow
TCGA-55-8089	702	1	56	MALE	unknow
TCGA-99-AA5R	658	0	70	FEMALE	unknow
TCGA-55-8505	440	0	62	MALE	unknow
TCGA-05-5423	151	0	65	MALE	unknow
TCGA-55-8614	536	0	76	MALE	unknow
TCGA-50-5946	1617	0	62	MALE	unknow
TCGA-05-4245	730	0	81	MALE	unknow
TCGA-91-A4BD	603	0	78	MALE	unknow
TCGA-55-8512	607	1	41	MALE	unknow
TCGA-NJ-A55A	15	0	76	FEMALE	unknow
TCGA-05-4434	457	1	67	FEMALE	unknow
TCGA-97-A4M6	568	0	45	FEMALE	unknow
TCGA-86-7954	605	0	68	FEMALE	unknow
TCGA-78-7150	666	1	59	MALE	unknow
TCGA-62-A470	1194	1	84	MALE	unknow
TCGA-69-8255	129	0	71	MALE	unknow
TCGA-55-8208	674	0	73	FEMALE	unknow
TCGA-50-5051	478	1	42	FEMALE	unknow
TCGA-55-7910	1040	0	50	FEMALE	unknow
TCGA-38-7271	800	1	72	FEMALE	unknow
TCGA-86-8585	353	0	57	MALE	unknow
TCGA-86-8075	694	1	66	FEMALE	unknow
TCGA-73-4676	281	1	45	MALE	unknow
TCGA-50-5933	2393	1	72	MALE	unknow
TCGA-73-4675	922	1	59	MALE	unknow
TCGA-55-6987	2137	0	77	MALE	unknow
TCGA-50-5055	1830	1	79	FEMALE	unknow
TCGA-MN-A4N5	84	0	63	MALE	unknow
TCGA-38-A44F	133	0	80	MALE	unknow
TCGA-55-6971	1400	0	59	FEMALE	unknow
TCGA-69-7974	184	0	54	FEMALE	unknow
TCGA-44-6779	500	1	50	FEMALE	unknow
TCGA-MP-A4TC	74	1	77	MALE	unknow
TCGA-55-8302	478	0	54	MALE	unknow
TCGA-67-3774	385	0	73	FEMALE	unknow
TCGA-55-A493	28	0	54	FEMALE	unknow
TCGA-50-7109	308	1	60	MALE	unknow
TCGA-55-6982	995	1	79	FEMALE	unknow

TCGA-86-8074	24	0	62	FEMALE	unknow
TCGA-69-A59K	591	0	60	FEMALE	unknow
TCGA-55-8090	598	1	80	MALE	unknow
TCGA-97-A4M2	624	0	66	MALE	unknow
TCGA-MP-A4T9	1265	1	54	FEMALE	unknow
TCGA-MP-A4TK	582	1	56	FEMALE	unknow
TCGA-91-6849	35	0	75	FEMALE	unknow
TCGA-50-6593	336	1	49	FEMALE	unknow
TCGA-95-7043	503	1	63	FEMALE	unknow
TCGA-55-7724	705	0	76	FEMALE	unknow
TCGA-53-A4EZ	1071	0	63	MALE	unknow
TCGA-44-7667	1097	0	49	FEMALE	unknow
TCGA-44-8117	385	0	54	FEMALE	unknow
TCGA-55-6983	2823	0	81	MALE	unknow
TCGA-44-6146	728	0	64	MALE	unknow
TCGA-55-8513	791	0	77	FEMALE	unknow
TCGA-MP-A5C7	2248	0	76	FEMALE	unknow
TCGA-75-6214	1115	1	unknow	FEMALE	unknow
TCGA-86-7713	1157	0	70	MALE	unknow
TCGA-75-6212	1516	1	unknow	FEMALE	unknow
TCGA-62-8394	139	1	65	FEMALE	unknow
TCGA-64-5778	1305	0	60	MALE	unknow
TCGA-97-8174	164	1	67	MALE	unknow
TCGA-50-8459	1119	0	68	MALE	unknow
TCGA-50-8457	1125	0	63	FEMALE	unknow
TCGA-97-A4M1	601	0	52	FEMALE	unknow
TCGA-78-7166	258	1	84	MALE	unknow
TCGA-83-5908	824	0	59	FEMALE	unknow
TCGA-05-4426	791	0	71	MALE	unknow
TCGA-55-7995	889	0	73	FEMALE	unknow
TCGA-93-8067	186	0	77	MALE	unknow
TCGA-55-7284	243	1	74	MALE	unknow
TCGA-55-1595	1479	0	74	FEMALE	unknow
TCGA-44-2655	1324	0	65	FEMALE	unknow
TCGA-69-8453	813	0	77	MALE	unknow
TCGA-91-6835	79	0	81	FEMALE	unknow
TCGA-95-8039	830	0	72	MALE	unknow
TCGA-97-7938	18	1	76	FEMALE	unknow
TCGA-69-7978	134	0	59	MALE	unknow
TCGA-55-7994	603	0	81	MALE	unknow
TCGA-55-8619	416	0	72	FEMALE	unknow
TCGA-78-7149	3940	0	71	MALE	unknow
TCGA-38-4631	354	1	72	FEMALE	unknow
TCGA-44-2662	1280	0	65	MALE	unknow
TCGA-MP-A4SV	2620	1	67	MALE	unknow
TCGA-L9-A7SV	565	0	69	MALE	unknow
TCGA-93-A4JQ	526	0	49	MALE	unknow
TCGA-35-3615	14	0	57	MALE	unknow
TCGA-05-4418	274	1	69	MALE	unknow
TCGA-05-4249	1523	0	67	MALE	unknow
TCGA-MN-A4N4	1175	0	57	MALE	unknow
TCGA-73-4658	1600	1	80	FEMALE	unknow
TCGA-44-A4SS	415	0	73	MALE	unknow

TCGA-95-A4VP	605	0	66	FEMALE	unknow
TCGA-55-7914	187	1	71	FEMALE	unknow
TCGA-78-7145	826	1	52	FEMALE	unknow
TCGA-55-8092	154	1	75	MALE	unknow
TCGA-MP-A4TE	896	1	56	MALE	unknow
TCGA-MP-A4T4	2617	1	68	FEMALE	unknow
TCGA-55-8203	547	0	69	FEMALE	unknow
TCGA-55-6712	171	1	71	MALE	unknow
TCGA-49-6743	1621	0	81	FEMALE	unknow
TCGA-73-4668	467	0	66	FEMALE	unknow
TCGA-67-3770	610	0	70	FEMALE	unknow
TCGA-64-1680	1126	0	63	MALE	unknow
TCGA-05-4398	1431	0	47	FEMALE	unknow
TCGA-78-7155	1171	1	68	MALE	unknow
TCGA-78-7152	1215	1	65	MALE	unknow
TCGA-75-6211		1	unknow	FEMALE	unknow
TCGA-MP-A4T6	1790	1	76	FEMALE	unknow
TCGA-55-A57B	546	0	80	FEMALE	unknow
TCGA-44-2656	1429	0	59	MALE	unknow
TCGA-93-A4JO	33	1	70	MALE	unknow
TCGA-78-7535	949	1	45	MALE	unknow
TCGA-62-8398	444	1	55	MALE	unknow
TCGA-91-6840	372	0	59	FEMALE	unknow
TCGA-75-5122		1	unknow	MALE	unknow
TCGA-99-8025	1060	0	72	FEMALE	unknow
TCGA-55-8207	977	0	73	MALE	unknow
TCGA-55-6969	1239	0	52	MALE	unknow
TCGA-64-5774	2676	0	60	MALE	unknow
TCGA-O1-A52J	1798	1	74	FEMALE	unknow
TCGA-86-6851	179	0	73	FEMALE	unknow
TCGA-62-8395	1216	0	80	FEMALE	unknow
TCGA-86-A4P7	415	0	63	FEMALE	unknow
TCGA-91-6831	310	0	66	MALE	unknow
TCGA-05-4420	912	0	41	MALE	unknow
TCGA-05-4395	0	1	76	MALE	unknow
TCGA-67-6217	422	0	73	FEMALE	unknow
TCGA-44-4112	808	1	60	FEMALE	unknow
TCGA-49-AAR2	2224	0	64	MALE	unknow
TCGA-62-A46O	1454	1	65	FEMALE	unknow
TCGA-44-7660	592	0	72	MALE	unknow
TCGA-44-3919	1026	1	71	FEMALE	unknow
TCGA-44-6775	705	0	72	FEMALE	unknow
TCGA-97-7554	775	0	83	FEMALE	unknow
TCGA-86-6562	376	1	52	MALE	unknow
TCGA-55-6642	2449	0	63	MALE	unknow
TCGA-62-A471	1246	0	64	MALE	unknow
TCGA-05-5715	62	0	69	FEMALE	unknow

stage	T	M	N
Stage IIA	T2a	MX	N1
Stage IA	T1b	MX	N0
Stage IV	T2	M1	N0
Stage IB	T2	M0	N0
Stage IV	T2	M1	N1
Stage IIIA	T2	M0	N2
Stage IB	T2	M0	N0
Stage IIA	T2a	M0	N1
Stage IV	T2	M1	N0
Stage IB	T2	M0	N0
Stage IIIA	T2	M0	N2
Stage IIB	T2	M0	N1
Stage IB	T2	MX	N0
Stage IIIA	T2a	MX	N2
Stage IA	T1	MX	N0
Stage IIIA	T2	M0	N2
Stage IIA	T2b	M0	N0
Stage IA	T2	M0	N0
Stage IIIA	T2	M0	N2
Stage IB	T2	M0	N0
Stage IB	T2	M0	N0
Stage IA	T1a	M0	N0
Stage IB	T2a	MX	N0
Stage IA	T1	M0	N0
Stage IB	T2a	MX	N0
Stage IB	T2a	MX	N0
Stage IA	T1b	MX	N0
Stage IIB	T3	MX	N0
Stage IIB	T3	M0	N0
Stage IB	T2a	unknow	N0
Stage IB	T2a	M0	N0
Stage IA	T1	M0	N0
Stage IA	T1a	M0	N0
Stage IA	T1	MX	N0
Stage IB	T2a	MX	N0
Stage IIA	T2b	M0	N0
Stage IB	T2a	MX	N0
Stage IB	T2a	M0	N0
Stage IA	T1	M0	NX
Stage IB	T2a	M0	N0
Stage IIA	T2a	M0	N1
Stage IV	T2	M1	N0
Stage IB	T2	M0	N0
Stage IIIA	T3	M0	N2
Stage IIIA	T2	M0	N2
Stage IA	T1b	MX	N0
Stage IIIB	T1a	MX	N3
Stage IIB	T2	M0	N1
Stage I	T1	M0	N0
Stage IIB	T2	M0	N1
Stage I	T1b	M0	N0

Stage IA	T1a	MX	N0
Stage IA	T1	M0	N0
Stage IA	T1	M0	N0
Stage IA	T1	M0	NX
Stage IIA	T2a	M0	N1
Stage IB	T2	M0	N0
Stage IB	T2	MX	N0
Stage IB	T2	M0	N0
Stage IA	T1b	MX	N0
Stage IA	T1	M0	N0
Stage IIB	T2	M0	N1
Stage I	T2	MX	N0
Stage IIIB	T4	M0	N2
Stage IIB	T3	M0	N0
Stage IV	T2	M1	N0
Stage IA	T1	M0	N0
Stage IB	T2	M0	N0
Stage IIIA	T2	M0	N2
Stage IIA	T2b	M0	N0
Stage IB	T2	M0	N0
Stage IIIA	T3	M0	N2
Stage IA	T1b	MX	N0
Stage IB	T2	M0	N0
Stage IB	T2	M0	N0
Stage IA	T1b	M0	N0
Stage IB	T2a	M0	N0
Stage IIB	T3	MX	N0
Stage IIIA	T2	M0	N2
Stage IB	T2	M0	N0
Stage IA	T1a	MX	N0
Stage IB	T2	M0	N0
Stage IB	T2	M0	N0
Stage IIA	T2a	M0	N1
Stage IIIA	T2	M0	N2
Stage IB	T2	M0	N0
Stage IB	T2	M0	N0
Stage IA	T1a	M0	N0
Stage IB	T2a	MX	N0
Stage IA	T1a	MX	N0
Stage IIA	T2b	M0	N0
Stage IB	T2	MX	N0
Stage IB	T2a	M0	N0
Stage IA	T1	M0	N0
Stage IIB	T2	M0	N1
Stage IB	T2a	M0	N0
Stage IIA	T1b	M0	N1
Stage IB	T2a	MX	N0
unknow	T4	MX	N0
Stage IIB	T3	M0	N0
Stage IA	T1	MX	N0
Stage IA	T1b	MX	N0
Stage IA	T1	M0	N0
Stage IA	T1	M0	N0

Stage IV	TX	M1	NX
Stage IIB	T2	MX	N1
Stage IIA	T2b	MX	N0
Stage IIA	T2b	MX	N0
Stage IIIA	T3	M0	N2
Stage IB	T2a	M0	N0
Stage IB	T2	M0	N0
Stage IA	T1b	M0	N0
Stage IIIA	T3	M0	N1
Stage IA	T1b	M0	N0
Stage IIIA	T2a	M0	N2
Stage IIA	T2a	M0	N1
Stage IIIA	T2	M0	N2
Stage IB	T2a	MX	N0
Stage IIB	T2	M0	N1
Stage IA	T1a	MX	N0
Stage IA	T1	M0	N0
Stage IIA	T2a	M0	N1
Stage IIA	T2a	M0	N1
Stage IA	T1a	MX	N0
Stage IV	T4	M1	N2
Stage IIB	T2	M0	N1
Stage IA	T1a	MX	N0
Stage IIIA	T3	MX	N2
Stage IV	T2	M1	N2
Stage IIIA	T2	MX	N2
Stage IIIA	T2	M0	N2
Stage IV	T1a	M1b	N1
Stage IB	T2	MX	N0
Stage IB	T2	MX	NX
Stage IIB	T3	M0	N0
Stage IA	T1a	MX	N0
Stage IA	T1a	M0	N0
Stage IIB	T3	M0	N0
Stage IIB	T2	M0	N1
Stage IA	T1a	M0	N0
Stage IA	T1b	M0	NX
Stage IIIA	T1a	MX	N2
Stage IIB	T3	MX	N0
Stage IIB	T2	M0	N1
Stage IB	T2	M0	N0
Stage IIB	T2	M0	N1
Stage IIA	T2a	M0	N1
Stage IB	T2a	MX	N0
Stage IA	T1	M0	N0
Stage IIIA	T1	M0	N2
Stage IA	T1a	M0	N0
Stage IA	T1b	MX	N0
Stage IA	T1	M0	N0
Stage IIB	T2b	M0	N1
Stage IA	T1b	MX	N0
Stage IIB	T2	M0	N1
Stage IB	T2a	M0	N0

Stage IIIA	T4	M0	N1
Stage IB	T2	M0	N0
Stage IA	T1b	M0	N0
Stage IIB	T3	MX	N0
Stage IA	T1	M0	N0
Stage IA	T1a	M0	N0
Stage IA	T1	unknow	NX
Stage IV	T2	M1	N0
Stage IB	T2	M0	N0
Stage IIIA	T1	M0	N2
Stage IA	T1a	MX	N0
Stage IIIB	T4	M0	N1
Stage IA	T1b	MX	N0
Stage IA	T1	MX	N0
Stage IA	T1b	M0	N0
Stage IA	T1a	MX	N0
Stage IIA	T1	MX	N1
Stage IIIA	T2b	unknow	N2
Stage IB	T2	MX	N0
Stage IA	T1a	MX	N0
Stage IIB	T3	M0	N0
Stage IA	T1a	M0	N0
Stage IA	T1a	MX	N0
unknow	T2b	M0	N0
Stage IIIA	T2	M0	N2
Stage IB	T2	MX	N0
Stage IB	T2a	M0	N0
Stage IA	T1	M0	N0
Stage IIIB	T4	M0	N1
Stage IA	T1b	M0	N0
Stage IA	T1a	MX	N0
Stage IIIA	T3	MX	N2
Stage IB	T2a	M0	N0
Stage IIA	T2a	M0	N1
unknow	T2b	M0	N0
Stage IA	T1a	M0	N0
Stage IIIA	T2	M0	N2
Stage IB	T2	M0	N0
Stage IIB	T3	M0	N0
Stage IA	T1b	M0	N0
Stage IIIA	T2	M0	N2
Stage IB	T2	M0	N0
Stage IA	T1	MX	N0
Stage IA	T1	M0	N0
Stage IIIA	T4	M0	N0
Stage IA	T1	M0	N0
Stage IB	T2a	MX	N0
Stage IA	T1b	M0	N0
Stage IB	T2a	MX	N0
Stage IIIA	T1b	M0	N2
Stage IB	T2	MX	N0
Stage IIIA	T2b	M0	N2
Stage IB	T2	M0	N0

Stage IA	T1	M0	N0
Stage IB	T2a	MX	N0
Stage IIIA	T1	M0	N2
Stage IIIA	T1b	MX	N2
Stage IIA	T2b	M0	N0
Stage IB	T2	M0	N0
Stage IB	T2a	M0	N0
Stage IIIA	T3	M0	N2
Stage IIB	T3	MX	N0
Stage IB	T2a	M0	N0
Stage IB	T2a	M0	N0
Stage IA	T1b	MX	N0
Stage IB	T2a	M0	N0
Stage IB	T2	M0	N0
Stage IIIA	T3	M0	N2
Stage I	T1	M0	N0
Stage IB	T2	M0	N0
Stage IIB	T2b	M0	N1
Stage IIIA	T3	M0	N1
Stage IIIA	T2	M0	N2
Stage IA	T1	M0	N0
Stage IIA	T1b	M0	N1
Stage IIA	T2a	M0	N1
Stage IA	T1b	MX	N0
Stage IA	T1	M0	N0
Stage IB	T2a	M0	N0
Stage IB	T2	M0	N0
Stage IB	T2	M0	N0
Stage IB	T2	M0	N0
Stage IIIA	T3	M0	N1
Stage IIIB	T4	M0	N2
Stage IIIB	T4	M0	N0
Stage IIIA	T2	MX	N2
Stage IIB	T3	M0	N0
Stage IA	T1b	M0	N0
Stage IV	T2b	M1b	N0
Stage IIA	T2a	M0	N1
unknow	T2b	unknow	unknow
Stage IIB	T2	MX	N1
Stage IIB	T2	M0	N1
Stage IA	T1b	M0	N0
Stage IA	T1b	M0	N0
Stage IIB	T2	M0	N1
Stage IA	T1b	M0	N0
Stage IA	T1b	M0	N0
Stage II	T1	MX	N1
Stage IB	T2	M0	N0
Stage IA	T1a	MX	N0
Stage IV	T2	M1	NX
unknow	T3	M0	N0
Stage IB	T2	M0	N0
Stage IIIA	T3	MX	N1
Stage IIIA	T2	M0	N2

Stage IIIA	T2	MX	N2
Stage IIIA	T2	M0	N2
Stage IIB	T2b	M0	N1
Stage IB	T2	M0	N0
Stage IB	T2a	M0	N0
Stage IIB	T2b	M0	N1
Stage IIA	T2a	MX	N1
Stage IA	T1	MX	N0
Stage IB	T2	M0	N0
Stage IIB	T2	M0	N1
Stage IA	T1	M0	N0
Stage IIIA	T2	M0	N2
Stage IIIA	T2	M0	N2
Stage IV	T2	M1	N0
Stage IB	T2a	M0	N0
Stage IIIA	T2	M0	N2
Stage IA	T1	M0	N0
Stage IIIA	T3	M0	N2
Stage IIA	T2a	MX	N1
Stage IIB	T2	M0	N1
unknow	T2a	M0	N0
Stage IB	T2a	M0	N0
Stage IB	T2	M0	N0
unknow	T2	M0	N1
Stage IA	T1a	MX	N0
Stage IB	T2a	MX	N0
Stage IIB	T2	M0	N1
Stage IA	T1b	MX	N0
Stage IA	T1	MX	N0
Stage IB	T2	M0	N0
Stage IA	T1b	MX	N0
Stage IA	T1	M0	N0
Stage IA	T1	M0	N0
Stage IIIA	T2	M0	N2
Stage IIIA	T4	M0	N0
Stage IA	T1a	MX	N0
Stage IIA	T1b	MX	N1
Stage IIB	T3	M0	N0
Stage IIIA	T2a	M0	N2
Stage IB	T2	M0	N0
Stage IV	T1	M1	N0
Stage IB	T2a	MX	N0
Stage IIA	T1a	MX	N1
Stage I	T1a	MX	N0
Stage IA	T1	MX	N0
Stage IV	T3	M1b	N2
Stage IA	T1b	M0	N0
Stage IB	T2	M0	N0
Stage IA	T1a	M0	N0
Stage IA	T1b	M0	N0
Stage IA	T1	M0	N0
Stage IB	T2	M0	N0
Stage IIB	T3	M0	N0

Stage IV	T1	M1	N0
Stage IB	T2a	MX	N0
Stage IB	T2	M0	N0
Stage IIB	T3	M0	N0
Stage IA	T1b	M0	N0
Stage IB	T2	M0	N0
Stage IA	T1	M0	N0
Stage IIB	T2b	M0	N1
Stage IB	T2a	M0	N0
Stage IV	T2a	M1a	N0
Stage IIB	T2	M0	N1
Stage IA	T1b	M0	N0
Stage IIIA	T2b	M0	N2
Stage IIA	T1b	M0	N1
Stage IB	T2a	M0	N0
Stage IIB	T2	M0	N1
Stage IIA	T2a	MX	N1
Stage IB	T2	M0	NX
Stage IV	T2a	M1a	N2
Stage IA	T1	M0	N0
Stage IB	T2	M0	N0
Stage IIIA	T4	M0	N0
Stage IIB	T3	MX	N0
Stage IIA	T2b	M0	N0
Stage IB	T2	MX	N0
Stage IA	T1a	M0	N0
Stage IB	T2	M0	N0
Stage IIA	T1b	MX	N1
Stage IIIA	T3	M0	N2
Stage IIIA	T2	M0	N2
Stage IIA	T1b	M0	N1
unknow	T1a	M0	N1
Stage IA	T1b	MX	N0
Stage IV	TX	MX	NX
Stage IV	T2	M1	N0
Stage IIB	T2	M0	N1
Stage IIB	T3	M0	N0
Stage IV	T4	M1	N1
Stage IIIA	T2	MX	N2
Stage IIIA	T2	M0	N2
Stage IB	T2a	M0	N0
Stage IB	T2	M0	N0
Stage IIA	T1	M0	N1
Stage IIB	T2	M0	N1
Stage IIB	T1	M0	N1
Stage IIB	T2	M0	N1
Stage IIA	T1b	M0	N1
Stage IA	T2	M0	N0
Stage IB	T2	M0	N0
Stage IA	T1	M0	N0
Stage IIA	T1	M0	N1
Stage IB	T2	M0	N0
Stage IA	T1a	M0	N0

Stage IIIA	T1	MX	N2
Stage IIB	T2	M0	N1
Stage IIA	T2b	MX	N0
Stage IA	T1a	MX	N0
Stage IA	T1	MX	N0
Stage IV	TX	M1b	NX
Stage IIB	T2	M0	N1
Stage IA	T1b	M0	N0
Stage IIIA	T2	M0	N2
Stage IB	T2	M0	N0
Stage IIB	T2	M0	N1
Stage IB	T2	M0	N0
Stage IB	T2a	MX	N0
Stage IB	T2a	M0	N0
Stage IB	T2a	MX	NX
Stage IA	T1a	M0	N0
Stage IA	T1a	M0	N0
Stage IIIA	T1a	MX	N2
Stage IIB	T2	M0	N1
Stage IB	T2a	MX	N0
Stage IA	T1	MX	N0
Stage IIIA	T2	M0	N2
Stage IIA	T1b	MX	N1
Stage IV	T1a	M1b	N1
Stage IB	T2	M0	N0
Stage IV	T4	M1	N1
Stage IA	T1a	M0	N0
Stage IB	T2	M0	N0
Stage IIB	T2	M0	N1
Stage IB	T2	M0	N0
Stage IA	T1a	M0	N0
Stage IA	T1b	M0	N0
Stage IIIA	T2	M0	N2
Stage IIA	T2b	M0	N0
Stage IA	T1	M0	N0
Stage IB	T2a	M0	N0
Stage IB	T2	M0	N0
Stage IIA	T2a	M0	N1
Stage IIIB	T4	M0	N2
Stage IIIA	T3	M0	N1
Stage IA	T1	M0	N0
Stage IIA	T1	M0	N1
Stage IA	T1a	M0	N0
Stage IB	T2a	M0	N0
Stage IB	T2	MX	N0
Stage IIIA	T2a	MX	N2
Stage IIB	T2	MX	N1
Stage IIIA	T1	M0	N2
Stage IB	T2	MX	N0
Stage IB	T2	M0	N0
Stage IB	T2a	M0	N0
Stage IA	T1	M0	N0
Stage IIB	T2	M0	N1

Stage IIA	T1b	M0	N1
Stage IIB	T3	M0	N0
Stage IA	T1a	M0	N0
Stage IA	T1a	M0	N0
Stage IIIA	T2	MX	N2
Stage IIB	T2	MX	N1
Stage IIIA	T2	MX	N2
Stage IIIA	T1	M0	N2
Stage IA	T1a	MX	N0
Stage IB	T2a	MX	N0
Stage IIA	T2a	MX	N1
Stage IIB	T3	MX	N0
Stage IB	T2a	M0	N0
Stage IIB	T2	M0	N1
Stage IIB	T3	M0	N0
Stage IIB	T3	MX	N0
Stage IB	T2	M0	N0
Stage IIIA	T2	M0	N2
Stage IIA	T2b	M0	N0
Stage IIB	T2	M0	N1
Stage IIIB	T4	M0	N2
Stage IB	T2	M0	N0
Stage IIA	T2b	M0	N0
Stage IIB	T3	M0	N0
Stage IA	T1a	M0	N0
Stage IA	T1a	M0	N0
Stage IIB	T2	M0	N1
Stage IA	T1	M0	N0
Stage IB	T2	M0	N0
Stage IA	T1b	M0	N0
Stage IB	T2a	MX	N0
Stage IIB	T3	MX	N0
Stage IA	T1	M0	N0
Stage IA	T1	M0	N0
Stage IIB	T3	MX	N0
Stage IA	T1	M0	N0
Stage IA	T1	MX	N0
Stage IA	T1a	MX	N0
Stage IIB	T2b	MX	N1
Stage IIB	T3	MX	N0
Stage IIB	T3	MX	N0
Stage IIIB	T4	M0	N0
Stage IB	T2	M0	N0
Stage IB	T2	M0	N0
Stage IB	T2	M0	N0
Stage IIA	T2a	M0	N1
Stage IA	T1b	MX	N0
Stage IB	T2	M0	N0
Stage IIIA	T3	M0	N2
Stage IB	T2	M0	N0
Stage IA	T1b	M0	N0
Stage IB	T2	M0	N0
Stage IA	T1b	M0	N0

Stage IIIA	T2b	M0	N2
Stage IIA	T1b	MX	N1
Stage IV	T4	M1	N1
Stage IIB	T3	MX	N0
Stage IIA	T2b	MX	N0
Stage IIB	T2	M0	N1
Stage IA	T1b	M0	N0
Stage IIA	T2a	MX	N1
Stage IIIA	T1	MX	N2
Stage IIB	T2	M0	N1
Stage IA	T1	M0	N0
Stage IV	T2a	M1	N2
Stage IIIB	T4	M0	N3
Stage IB	T2	M0	N0
Stage IB	T2	M0	N0
Stage IB	T2	M0	N0
Stage IIIA	T1	MX	N2
Stage IA	T1b	M0	N0
Stage IB	T2	M0	N0
Stage IA	T1a	MX	N0
Stage IB	T2	M0	N0
Stage IIIA	T2	M0	N2
Stage IA	T1b	M0	N0
Stage IB	T2	M0	N0
Stage IIIA	T3	M0	N2
Stage IB	T2a	MX	N0
Stage IB	T2	M0	N0
Stage IB	T2	M0	N0
Stage IA	T1	MX	N0
Stage IIA	T1b	M0	N1
Stage IIB	T3	M0	N0
Stage IB	T2a	M0	N0
Stage IB	T2	MX	N0
Stage IB	T2	M0	N0
Stage IIIB	T4	M0	N2
Stage IIA	T2a	M0	N1
Stage IB	T2a	M0	N0
Stage IB	T2	MX	N0
Stage IB	T2	M0	N0
Stage IB	T2	MX	N0
Stage IA	T1	M0	N0
Stage IB	T2a	MX	N0
Stage IIIA	T2a	M0	N2
Stage IIA	T2a	M0	N1
Stage IB	T2	MX	N0
Stage IIB	T2b	M0	N1
Stage IB	T2a	M0	N0
