# Supplementary material for: Integrative Molecular Analyses of an Individual Transcription Factor-Based Genomic Model for Lung Cancer Prognosis
Source: Dis Markers. 2021 Dec 7;2021:5125643. doi: 10.1155/2021/5125643 (PMC8672105; doi:10.1155/2021/5125643)
Supplement: Supplementary 2 — Supplementary Table 2: the gene sets of TFs. [file 5125643.f2.pdf]

Supplementary table 2. The gene sets of TFs.
AC008770.3
AC023509.3
AC092835.1
AC138696.1
ADNP
ADNP2
AEBP1
AEBP2
AHCTF1
AHDC1
AHR
AHRR
AIRE
AKAP8
AKAP8L
AKNA
ALX1
ALX3
ALX4
ANHX
ANKZF1
AR
ARGFX
ARHGAP35
ARID2
ARID3A
ARID3B
ARID3C
ARID5A
ARID5B
ARNT
ARNT2
ARNTL
ARNTL2
ARX
ASCL1
ASCL2
ASCL3
ASCL4
ASCL5
ASH1L
ATF1
ATF2
ATF3
ATF4
ATF5
ATF6
ATF6B
ATF7
ATMIN
ATOH1
ATOH7

ATOH8
BACH1
BACH2
BARHL1
BARHL2
BARX1
BARX2
BATF
BATF2
BATF3
BAZ2A
BAZ2B
BBX
BCL11A
BCL11B
BCL6
BCL6B
BHLHA15
BHLHA9
BHLHE22
BHLHE23
BHLHE40
BHLHE41
BNC1
BNC2
BORCS8-MEF2B
BPTF
BRF2
BSX
C11orf95
CAMTA1
CAMTA2
CARF
CASZ1
CBX2
CC2D1A
CCDC169-SOHLH2
CCDC17
CDC5L
CDX1
CDX2
CDX4
CEBPA
CEBPB
CEBPD
CEBPE
CEBPG
CEBPZ
CENPA
CENPB
CENPBD1
CENPS
CENPT

CENPX
CGGBP1
CHAMP1
CHCHD3
CIC
CLOCK
CPEB1
CPXCR1
CREB1
CREB3
CREB3L1
CREB3L2
CREB3L3
CREB3L4
CREB5
CREBL2
CREBZF
CREM
CRX
CSRNP1
CSRNP2
CSRNP3
CTCF
CTCFL
CUX1
CUX2
CXXC1
CXXC4
CXXC5
DACH1
DACH2
DBP
DBX1
DBX2
DDIT3
DEAF1
DLX1
DLX2
DLX3
DLX4
DLX5
DLX6
DMBX1
DMRT1
DMRT2
DMRT3
DMRTA1
DMRTA2
DMRTB1
DMRTC2
DMTF1
DNMT1
DNTTIP1

DOT1L
DPF1
DPF3
DPRX
DR1
DRAP1
DRGX
DUX1
DUX3
DUX4
DUXA
DZIP1
E2F1
E2F2
E2F3
E2F4
E2F5
E2F6
E2F7
E2F8
E4F1
EBF1
EBF2
EBF3
EBF4
EEA1
EGR1
EGR2
EGR3
EGR4
EHF
ELF1
ELF2
ELF3
ELF4
ELF5
ELK1
ELK3
ELK4
EMX1
EMX2
EN1
EN2
EOMES
EPAS1
ERF
ERG
ESR1
ESR2
ESRRA
ESRRB
ESRRG
ESX1

ETS1
ETS2
ETV1
ETV2
ETV3
ETV3L
ETV4
ETV5
ETV6
ETV7
EVX1
EVX2
FAM170A
FAM200B
FBXL19
FERD3L
FEV
FEZF1
FEZF2
FIGLA
FIZ1
FLI1
FLYWCH1
FOS
FOSB
FOSL1
FOSL2
FOXA1
FOXA2
FOXA3
FOXB1
FOXB2
FOXC1
FOXC2
FOXD1
FOXD2
FOXD3
FOXD4
FOXD4L1
FOXD4L3
FOXD4L4
FOXD4L5
FOXD4L6
FOXE1
FOXE3
FOXF1
FOXF2
FOXG1
FOXH1
FOXI1
FOXI2
FOXI3
FOXJ1

FOXJ2
FOXJ3
FOXK1
FOXK2
FOXL1
FOXL2
FOXM1
FOXN1
FOXN2
FOXN3
FOXN4
FOXO1
FOXO3
FOXO4
FOXO6
FOXP1
FOXP2
FOXP3
FOXP4
FOXQ1
FOXR1
FOXR2
FOXS1
GABPA
GATA1
GATA2
GATA3
GATA4
GATA5
GATA6
GATAD2A
GATAD2B
GBX1
GBX2
GCM1
GCM2
GFI1
GFI1B
GLI1
GLI2
GLI3
GLI4
GLIS1
GLIS2
GLIS3
GLMP
GLYR1
GMEB1
GMEB2
GPBP1
GPBP1L1
GRHL1
GRHL2

GRHL3
GSC
GSC2
GSX1
GSX2
GTF2B
GTF2I
GTF2IRD1
GTF2IRD2
GTF2IRD2B
GTF3A
GZF1
HAND1
HAND2
HBP1
HDX
HELT
HES1
HES2
HES3
HES4
HES5
HES6
HES7
HESX1
HEY1
HEY2
HEYL
HHEX
HIC1
HIC2
HIF1A
HIF3A
HINFP
HIVEP1
HIVEP2
HIVEP3
HKR1
HLF
HLX
HMBOX1
HMG20A
HMG20B
HMGA1
HMGA2
HMGN3
HMX1
HMX2
HMX3
HNF1A
HNF1B
HNF4A
HNF4G

HOMEZ
HOXA1
HOXA10
HOXA11
HOXA13
HOXA2
HOXA3
HOXA4
HOXA5
HOXA6
HOXA7
HOXA9
HOXB1
HOXB13
HOXB2
HOXB3
HOXB4
HOXB5
HOXB6
HOXB7
HOXB8
HOXB9
HOXC10
HOXC11
HOXC12
HOXC13
HOXC4
HOXC5
HOXC6
HOXC8
HOXC9
HOXD1
HOXD10
HOXD11
HOXD12
HOXD13
HOXD3
HOXD4
HOXD8
HOXD9
HSF1
HSF2
HSF4
HSF5
HSFX1
HSFX2
HSFY1
HSFY2
IKZF1
IKZF2
IKZF3
IKZF4
IKZF5

INSM1
INSM2
IRF1
IRF2
IRF3
IRF4
IRF5
IRF6
IRF7
IRF8
IRF9
IRX1
IRX2
IRX3
IRX4
IRX5
IRX6
ISL1
ISL2
ISX
JAZF1
JDP2
JRK
JRKL
JUN
JUNB
JUND
KAT7
KCMF1
KCNIP3
KDM2A
KDM2B
KDM5B
KIN
KLF1
KLF10
KLF11
KLF12
KLF13
KLF14
KLF15
KLF16
KLF17
KLF2
KLF3
KLF4
KLF5
KLF6
KLF7
KLF8
KLF9
KMT2A
KMT2B

L3MBTL1
L3MBTL3
L3MBTL4
LBX1
LBX2
LCOR
LCORL
LEF1
LEUTX
LHX1
LHX2
LHX3
LHX4
LHX5
LHX6
LHX8
LHX9
LIN28A
LIN28B
LIN54
LMX1A
LMX1B
LTF
LYL1
MAF
MAFA
MAFB
MAFF
MAFG
MAFK
MAX
MAZ
MBD1
MBD2
MBD3
MBD4
MBD6
MBNL2
MECOM
MECP2
MEF2A
MEF2B
MEF2C
MEF2D
MEIS1
MEIS2
MEIS3
MEOX1
MEOX2
MESP1
MESP2
MGA
MITF

MIXL1
MKX
MLX
MLXIP
MLXIPL
MNT
MNX1
MSANTD1
MSANTD3
MSANTD4
MSC
MSGN1
MSX1
MSX2
MTERF1
MTERF2
MTERF3
MTERF4
MTF1
MTF2
MXD1
MXD3
MXD4
MXI1
MYB
MYBL1
MYBL2
MYC
MYCL
MYCN
MYF5
MYF6
MYNN
MYOD1
MYOG
MYPOP
MYRF
MYRFL
MYSM1
MYT1
MYT1L
MZF1
NACC2
NAIF1
NANOG
NANOGNB
NANOGP8
NCOA1
NCOA2
NCOA3
NEUROD1
NEUROD2
NEUROD4

NEUROD6
NEUROG1
NEUROG2
NEUROG3
NFAT5
NFATC1
NFATC2
NFATC3
NFATC4
NFE2
NFE2L1
NFE2L2
NFE2L3
NFE4
NFIA
NFIB
NFIC
NFIL3
NFIX
NFKB1
NFKB2
NFX1
NFXL1
NFYA
NFYB
NFYC
NHLH1
NHLH2
NKRF
NKX1-1
NKX1-2
NKX2-1
NKX2-2
NKX2-3
NKX2-4
NKX2-5
NKX2-6
NKX2-8
NKX3-1
NKX3-2
NKX6-1
NKX6-2
NKX6-3
NME2
NOBOX
NOTO
NPAS1
NPAS2
NPAS3
NPAS4
NR0B1
NR1D1
NR1D2

NR1H2
NR1H3
NR1H4
NR1I2
NR1I3
NR2C1
NR2C2
NR2E1
NR2E3
NR2F1
NR2F2
NR2F6
NR3C1
NR3C2
NR4A1
NR4A2
NR4A3
NR5A1
NR5A2
NR6A1
NRF1
NRL
OLIG1
OLIG2
OLIG3
ONECUT1
ONECUT2
ONECUT3
OSR1
OSR2
OTP
OTX1
OTX2
OVOL1
OVOL2
OVOL3
PA2G4
PATZ1
PAX1
PAX2
PAX3
PAX4
PAX5
PAX6
PAX7
PAX8
PAX9
PBX1
PBX2
PBX3
PBX4
PCGF2
PCGF6

PDX1
PEG3
PGR
PHF1
PHF19
PHF20
PHF21A
PHOX2A
PHOX2B
PIN1
PITX1
PITX2
PITX3
PKNOX1
PKNOX2
PLAG1
PLAGL1
PLAGL2
PLSCR1
POGK
POU1F1
POU2AF1
POU2F1
POU2F2
POU2F3
POU3F1
POU3F2
POU3F3
POU3F4
POU4F1
POU4F2
POU4F3
POU5F1
POU5F1B
POU5F2
POU6F1
POU6F2
PPARA
PPARD
PPARG
PRDM1
PRDM10
PRDM12
PRDM13
PRDM14
PRDM15
PRDM16
PRDM2
PRDM4
PRDM5
PRDM6
PRDM8
PRDM9

PREB
PRMT3
PROP1
PROX1
PROX2
PRR12
PRRX1
PRRX2
PTF1A
PURA
PURB
PURG
RAG1
RARA
RARB
RARG
RAX
RAX2
RBAK
RBCK1
RBPJ
RBPJL
RBSN
REL
RELA
RELB
REPIN1
REST
REXO4
RFX1
RFX2
RFX3
RFX4
RFX5
RFX6
RFX7
RFX8
RHOXF1
RHOXF2
RHOXF2B
RLF
RORA
RORB
RORC
RREB1
RUNX1
RUNX2
RUNX3
RXRA
RXRB
RXRG
SAFB
SAFB2

SALL1
SALL2
SALL3
SALL4
SATB1
SATB2
SCMH1
SCML4
SCRT1
SCRT2
SCX
SEBOX
SETBP1
SETDB1
SETDB2
SGSM2
SHOX
SHOX2
SIM1
SIM2
SIX1
SIX2
SIX3
SIX4
SIX5
SIX6
SKI
SKIL
SKOR1
SKOR2
SLC2A4RG
SMAD1
SMAD3
SMAD4
SMAD5
SMAD9
SMYD3
SNAI1
SNAI2
SNAI3
SNAPC2
SNAPC4
SNAPC5
SOHLH1
SOHLH2
SON
SOX1
SOX10
SOX11
SOX12
SOX13
SOX14
SOX15

SOX17
SOX18
SOX2
SOX21
SOX3
SOX30
SOX4
SOX5
SOX6
SOX7
SOX8
SOX9
SP1
SP100
SP110
SP140
SP140L
SP2
SP3
SP4
SP5
SP6
SP7
SP8
SP9
SPDEF
SPEN
SPI1
SPIB
SPIC
SPZ1
SRCAP
SREBF1
SREBF2
SRF
SRY
ST18
STAT1
STAT2
STAT3
STAT4
STAT5A
STAT5B
STAT6
T
TAL1
TAL2
TBP
TBPL1
TBPL2
TBR1
TBX1
TBX10

TBX15
TBX18
TBX19
TBX2
TBX20
TBX21
TBX22
TBX3
TBX4
TBX5
TBX6
TCF12
TCF15
TCF20
TCF21
TCF23
TCF24
TCF3
TCF4
TCF7
TCF7L1
TCF7L2
TCFL5
TEAD1
TEAD2
TEAD3
TEAD4
TEF
TERB1
TERF1
TERF2
TET1
TET2
TET3
TFAP2A
TFAP2B
TFAP2C
TFAP2D
TFAP2E
TFAP4
TFCP2
TFCP2L1
TFDP1
TFDP2
TFDP3
TFE3
TFEB
TFEC
TGIF1
TGIF2
TGIF2LX
TGIF2LY
THAP1

THAP10
THAP11
THAP12
THAP2
THAP3
THAP4
THAP5
THAP6
THAP7
THAP8
THAP9
THRA
THRB
THYN1
TIGD1
TIGD2
TIGD3
TIGD4
TIGD5
TIGD6
TIGD7
TLX1
TLX2
TLX3
TMF1
TOPORS
TP53
TP63
TP73
TPRX1
TRAFD1
TRERF1
TRPS1
TSC22D1
TSHZ1
TSHZ2
TSHZ3
TTF1
TWIST1
TWIST2
UBP1
UNCX
USF1
USF2
USF3
VAX1
VAX2
VDR
VENTX
VEZF1
VSX1
VSX2
WIZ

WT1
XBP1
XPA
YBX1
YBX2
YBX3
YY1
YY2
ZBED1
ZBED2
ZBED3
ZBED4
ZBED5
ZBED6
ZBED9
ZBTB1
ZBTB10
ZBTB11
ZBTB12
ZBTB14
ZBTB16
ZBTB17
ZBTB18
ZBTB2
ZBTB20
ZBTB21
ZBTB22
ZBTB24
ZBTB25
ZBTB26
ZBTB3
ZBTB32
ZBTB33
ZBTB34
ZBTB37
ZBTB38
ZBTB39
ZBTB4
ZBTB40
ZBTB41
ZBTB42
ZBTB43
ZBTB44
ZBTB45
ZBTB46
ZBTB47
ZBTB48
ZBTB49
ZBTB5
ZBTB6
ZBTB7A
ZBTB7B
ZBTB7C

ZBTB8A
ZBTB8B
ZBTB9
ZC3H8
ZEB1
ZEB2
ZFAT
ZFHX2
ZFHX3
ZFHX4
ZFP1
ZFP14
ZFP2
ZFP28
ZFP3
ZFP30
ZFP37
ZFP41
ZFP42
ZFP57
ZFP62
ZFP64
ZFP69
ZFP69B
ZFP82
ZFP90
ZFP91
ZFP92
ZFPM1
ZFPM2
ZFX
ZFY
ZGLP1
ZGPAT
ZHX1
ZHX2
ZHX3
ZIC1
ZIC2
ZIC3
ZIC4
ZIC5
ZIK1
ZIM2
ZIM3
ZKSCAN1
ZKSCAN2
ZKSCAN3
ZKSCAN4
ZKSCAN5
ZKSCAN7
ZKSCAN8
ZMAT1

ZMAT4
ZNF10
ZNF100
ZNF101
ZNF107
ZNF112
ZNF114
ZNF117
ZNF12
ZNF121
ZNF124
ZNF131
ZNF132
ZNF133
ZNF134
ZNF135
ZNF136
ZNF138
ZNF14
ZNF140
ZNF141
ZNF142
ZNF143
ZNF146
ZNF148
ZNF154
ZNF155
ZNF157
ZNF16
ZNF160
ZNF165
ZNF169
ZNF17
ZNF174
ZNF175
ZNF177
ZNF18
ZNF180
ZNF181
ZNF182
ZNF184
ZNF189
ZNF19
ZNF195
ZNF197
ZNF2
ZNF20
ZNF200
ZNF202
ZNF205
ZNF207
ZNF208
ZNF211

ZNF212
ZNF213
ZNF214
ZNF215
ZNF217
ZNF219
ZNF22
ZNF221
ZNF222
ZNF223
ZNF224
ZNF225
ZNF226
ZNF227
ZNF229
ZNF23
ZNF230
ZNF232
ZNF233
ZNF234
ZNF235
ZNF236
ZNF239
ZNF24
ZNF248
ZNF25
ZNF250
ZNF251
ZNF253
ZNF254
ZNF256
ZNF257
ZNF26
ZNF260
ZNF263
ZNF264
ZNF266
ZNF267
ZNF268
ZNF273
ZNF274
ZNF275
ZNF276
ZNF277
ZNF28
ZNF280A
ZNF280B
ZNF280C
ZNF280D
ZNF281
ZNF282
ZNF283
ZNF284

ZNF285
ZNF286A
ZNF286B
ZNF287
ZNF292
ZNF296
ZNF3
ZNF30
ZNF300
ZNF302
ZNF304
ZNF311
ZNF316
ZNF317
ZNF318
ZNF319
ZNF32
ZNF320
ZNF322
ZNF324
ZNF324B
ZNF326
ZNF329
ZNF331
ZNF333
ZNF334
ZNF335
ZNF337
ZNF33A
ZNF33B
ZNF34
ZNF341
ZNF343
ZNF345
ZNF346
ZNF347
ZNF35
ZNF350
ZNF354A
ZNF354B
ZNF354C
ZNF358
ZNF362
ZNF365
ZNF366
ZNF367
ZNF37A
ZNF382
ZNF383
ZNF384
ZNF385A
ZNF385B
ZNF385C

ZNF385D
ZNF391
ZNF394
ZNF395
ZNF396
ZNF397
ZNF398
ZNF404
ZNF407
ZNF408
ZNF41
ZNF410
ZNF414
ZNF415
ZNF416
ZNF417
ZNF418
ZNF419
ZNF420
ZNF423
ZNF425
ZNF426
ZNF428
ZNF429
ZNF43
ZNF430
ZNF431
ZNF432
ZNF433
ZNF436
ZNF438
ZNF439
ZNF44
ZNF440
ZNF441
ZNF442
ZNF443
ZNF444
ZNF445
ZNF446
ZNF449
ZNF45
ZNF451
ZNF454
ZNF460
ZNF461
ZNF462
ZNF467
ZNF468
ZNF469
ZNF470
ZNF471
ZNF473

ZNF474
ZNF479
ZNF48
ZNF480
ZNF483
ZNF484
ZNF485
ZNF486
ZNF487
ZNF488
ZNF490
ZNF491
ZNF492
ZNF493
ZNF496
ZNF497
ZNF500
ZNF501
ZNF502
ZNF503
ZNF506
ZNF507
ZNF510
ZNF511
ZNF512
ZNF512B
ZNF513
ZNF514
ZNF516
ZNF517
ZNF518A
ZNF518B
ZNF519
ZNF521
ZNF524
ZNF525
ZNF526
ZNF527
ZNF528
ZNF529
ZNF530
ZNF532
ZNF534
ZNF536
ZNF540
ZNF541
ZNF543
ZNF544
ZNF546
ZNF547
ZNF548
ZNF549
ZNF550

ZNF551
ZNF552
ZNF554
ZNF555
ZNF556
ZNF557
ZNF558
ZNF559
ZNF560
ZNF561
ZNF562
ZNF563
ZNF564
ZNF565
ZNF566
ZNF567
ZNF568
ZNF569
ZNF57
ZNF570
ZNF571
ZNF572
ZNF573
ZNF574
ZNF575
ZNF576
ZNF577
ZNF578
ZNF579
ZNF580
ZNF581
ZNF582
ZNF583
ZNF584
ZNF585A
ZNF585B
ZNF586
ZNF587
ZNF587B
ZNF589
ZNF592
ZNF594
ZNF595
ZNF596
ZNF597
ZNF598
ZNF599
ZNF600
ZNF605
ZNF606
ZNF607
ZNF608
ZNF609

ZNF610
ZNF611
ZNF613
ZNF614
ZNF615
ZNF616
ZNF618
ZNF619
ZNF620
ZNF621
ZNF623
ZNF624
ZNF625
ZNF626
ZNF627
ZNF628
ZNF629
ZNF630
ZNF639
ZNF641
ZNF644
ZNF645
ZNF646
ZNF648
ZNF649
ZNF652
ZNF653
ZNF654
ZNF655
ZNF658
ZNF66
ZNF660
ZNF662
ZNF664
ZNF665
ZNF667
ZNF668
ZNF669
ZNF670
ZNF671
ZNF672
ZNF674
ZNF675
ZNF676
ZNF677
ZNF678
ZNF679
ZNF680
ZNF681
ZNF682
ZNF683
ZNF684
ZNF687

ZNF688
ZNF689
ZNF69
ZNF691
ZNF692
ZNF695
ZNF696
ZNF697
ZNF699
ZNF7
ZNF70
ZNF700
ZNF701
ZNF703
ZNF704
ZNF705A
ZNF705B
ZNF705D
ZNF705E
ZNF705G
ZNF706
ZNF707
ZNF708
ZNF709
ZNF71
ZNF710
ZNF711
ZNF713
ZNF714
ZNF716
ZNF717
ZNF718
ZNF721
ZNF724
ZNF726
ZNF727
ZNF728
ZNF729
ZNF730
ZNF732
ZNF735
ZNF736
ZNF737
ZNF74
ZNF740
ZNF746
ZNF747
ZNF749
ZNF750
ZNF75A
ZNF75D
ZNF76
ZNF761

ZNF763
ZNF764
ZNF765
ZNF766
ZNF768
ZNF77
ZNF770
ZNF771
ZNF772
ZNF773
ZNF774
ZNF775
ZNF776
ZNF777
ZNF778
ZNF780A
ZNF780B
ZNF781
ZNF782
ZNF783
ZNF784
ZNF785
ZNF786
ZNF787
ZNF788
ZNF789
ZNF79
ZNF790
ZNF791
ZNF792
ZNF793
ZNF799
ZNF8
ZNF80
ZNF800
ZNF804A
ZNF804B
ZNF805
ZNF808
ZNF81
ZNF813
ZNF814
ZNF816
ZNF821
ZNF823
ZNF827
ZNF829
ZNF83
ZNF830
ZNF831
ZNF835
ZNF836
ZNF837

ZNF84
ZNF841
ZNF843
ZNF844
ZNF845
ZNF846
ZNF85
ZNF850
ZNF852
ZNF853
ZNF860
ZNF865
ZNF878
ZNF879
ZNF880
ZNF883
ZNF888
ZNF891
ZNF90
ZNF91
ZNF92
ZNF93
ZNF98
ZNF99
ZSCAN1
ZSCAN10
ZSCAN12
ZSCAN16
ZSCAN18
ZSCAN2
ZSCAN20
ZSCAN21
ZSCAN22
ZSCAN23
ZSCAN25
ZSCAN26
ZSCAN29
ZSCAN30
ZSCAN31
ZSCAN32
ZSCAN4
ZSCAN5A
ZSCAN5B
ZSCAN5C
ZSCAN9
ZUFSP
ZXDA
ZXDB
ZXDC
ZZZ3
