# Supplementary material for: Integrative Molecular Analyses of an Individual Transcription Factor-Based Genomic Model for Lung Cancer Prognosis
Source: Dis Markers. 2021 Dec 7;2021:5125643. doi: 10.1155/2021/5125643 (PMC8672105; doi:10.1155/2021/5125643)
Supplement: Supplementary 3 — Supplementary Table 3: the detailed information of lung cancer-specific TFs. [file 5125643.f3.pdf]

Supplementary table 3. The detailed information of lung cancer-specific TFs.

ID	baseMean	log2FoldChange	lfcSE
EPAS1	27384.76111	-2.71284645	0.109800312
ETV4	2125.944896	3.839676614	0.158093613
E2F8	346.5473599	3.173793034	0.149159771
PITX2	330.8573005	8.205290079	0.394250299
FOXF1	599.8446709	-2.732969698	0.13457612
TAL1	192.8670911	-2.776906928	0.137598228
CENPA	283.3774826	3.626518426	0.182258949
FOXM1	1548.891696	3.361626424	0.171049208
MYBL2	2279.403521	3.811707506	0.19773459
ERG	1006.537774	-2.136957853	0.111524785
CSRNP1	3305.511999	-2.379870092	0.124558263
OTX1	268.5897869	3.322371502	0.175940834
MNX1	116.2723098	4.324482312	0.232659935
SOX7	354.0719024	-2.929434769	0.158834742
TFAP2A	716.9181753	3.736199613	0.203225959
GRHL3	158.8026345	3.511605084	0.196790545
SOX17	169.1511492	-2.700114114	0.152085533
FEZF1	163.1468312	5.640125896	0.321536494
BARX1	698.3252289	7.541027306	0.430136819
SALL4	119.3825944	3.424407887	0.196938181
DMBX1	73.32707563	6.491072326	0.374071057
ONECUT1	43.0296216	6.693296757	0.386474284
PITX1	691.4609906	4.532655905	0.263733033
HNF4G	401.465236	3.51290157	0.205564172
YBX2	257.3408165	4.747388089	0.278290982
KLF6	10185.68744	-1.785156011	0.107234023
ONECUT2	211.2681111	4.95203031	0.305448991
JDP2	1582.66599	-1.457919492	0.090464096
BCL6B	744.7656358	-1.764376764	0.109492069
E2F2	313.312442	2.341886447	0.14594714
SOX4	10616.50558	1.660777193	0.103618389
TCF21	524.6782549	-3.089978936	0.193537394
E2F3	1335.062886	1.367790398	0.086037109
NKX1-2	95.03151789	7.645214159	0.480951881
TBX15	477.4579687	3.738254869	0.235757335
BARX2	501.075161	3.872219921	0.244352193
ZNF695	53.53588116	4.459676831	0.283421362
SPDEF	1830.883843	3.265665896	0.208322324
FLI1	1243.823863	-1.670569475	0.109368189
HOXB9	341.9024662	6.649902781	0.436638391
FOXI3	130.9375278	6.811094821	0.448478663
HMGA1	11839.12917	2.269674069	0.150056454
LBX2	66.3752348	2.112126583	0.13974054
KLF13	5104.710105	-1.381100719	0.091686114
HMGA2	792.9281143	5.753282068	0.382179847
TFAP4	690.0066239	1.233686791	0.082378854
TBX3	691.1332587	-2.11343544	0.141777476
ZFP69B	110.0642211	1.691856219	0.114550194
PDX1	57.18484619	8.985259778	0.609074079
ZNF239	278.1253161	1.681533065	0.115504121
E2F7	198.5564913	2.72874332	0.188763854

PKNOX2	235.9440601	-2.424463482	0.169266946
KLF9	3186.679182	-1.662823838	0.116225011
SIX4	821.3075585	1.918419139	0.134547019
SP8	100.5019094	6.908053244	0.485064295
TBX2	1491.236387	-1.897490769	0.133661638
KDM5B	5438.014844	1.074778997	0.076391099
HOXC13	94.5274958	7.092391518	0.509292914
ZNF366	118.131607	-1.95127883	0.142008954
ZNF485	148.5145013	1.235646782	0.090011006
ZIC2	81.04072097	5.232596563	0.38294723
GATA2	743.7388768	-1.86308419	0.136540117
POU3F2	98.93812289	5.866769186	0.430205897
KLF4	2227.656797	-2.273128912	0.166957338
PAX7	402.5025073	7.886115929	0.579237153
DACH1	267.1414602	-2.459890604	0.18310552
DMRTA2	104.3666485	5.077060909	0.378022021
ZNF692	1341.420374	1.627182256	0.121445186
FOXP3	257.9091787	1.844907136	0.138122473
MAFF	1759.238988	-1.521451072	0.114144106
HOXA10	149.7947787	4.210807637	0.316211037
HOXC11	121.6423215	6.932294704	0.521422103
BHLHA15	140.8346487	2.88492639	0.217021525
HOXC10	299.4429535	6.051357585	0.45537937
ZNF423	210.1027672	-1.802923692	0.137147246
TBX4	614.9629199	-2.176240516	0.165828757
JUND	7220.621565	-1.450750841	0.111334788
KLF10	3831.920525	-1.13596299	0.087232732
MESP1	194.8943237	2.377652251	0.182740445
OVOL1	242.130585	2.311921562	0.178335621
LHX2	29.42837585	4.724193125	0.365778643
MESP2	59.6022031	3.131904434	0.242995221
ZBTB4	5515.616964	-1.018476442	0.07907597
CBX2	819.281614	2.491067244	0.193477385
POU4F1	41.45992821	5.197647575	0.404284341
FOXA3	389.0862591	3.28315171	0.25676406
HES6	1051.943609	2.840779177	0.222195507
RUNX2	875.9381714	1.626106114	0.127364354
EGR2	840.6644785	-1.867015546	0.146524034
ZNF217	4183.662322	1.334823959	0.105166739
ARNTL2	1644.459592	2.487227382	0.196517336
ZBED2	301.8903528	-2.356711109	0.18623824
ZBTB47	712.2048851	-1.085340905	0.085877237
RFX2	810.8122536	-1.623855309	0.128894489
HOXB13	121.9542384	6.507903578	0.517391529
ZNF138	469.6021026	1.193343098	0.094996687
FOXB1	53.71532568	4.502302399	0.363858987
SOX11	152.3812301	4.29955044	0.348216074
SHOX2	88.56365413	3.174844241	0.257198797
CREB3L4	1179.184675	1.387374644	0.112394218
FBXL19	1180.50829	1.189131476	0.096413291
SOX18	370.1250024	-1.638693035	0.134461098
SOHLH2	52.28568008	4.492591613	0.369116646
TFAP2D	57.27765258	6.985681945	0.584903894

HOXA5	299.0343487	-1.602118029	0.134923344
SALL1	83.92764463	5.741278511	0.4854042
ZNF107	726.4482673	1.322414492	0.11189336
SIX1	1245.542451	2.359801116	0.200439437
FOSB	8677.020491	-3.287144006	0.279935187
VSX1	18.95102367	3.906353572	0.334673208
SPI1	2280.146517	-1.586699573	0.136371055
SOX12	1549.973279	1.340594596	0.116065781
ZEB1	1286.885165	-1.333573078	0.115880842
KLF2	1460.893741	-1.733380373	0.150752516
ZNF92	585.4806121	1.03555921	0.091106923
EN2	33.39724703	4.192317801	0.369510354
DLX6	43.97965823	5.027869641	0.443187436
PAX9	652.3707246	2.699847464	0.238064754
FOXO6	213.3062367	2.268090984	0.200873786
SIX2	288.0926749	3.281369066	0.290948754
E2F5	759.0698987	1.262777058	0.112056
ARID5A	1326.249399	-1.085239326	0.096665898
FOXE1	143.713847	4.243662734	0.381935749
TBX5	474.0074332	-1.485710398	0.133875974
NFATC1	885.0962272	-1.144878921	0.10323913
MXD3	345.1283191	1.379383861	0.125049565
HNF1A	104.0965995	3.827158198	0.347787432
HOXC9	72.86647232	3.293371424	0.301692946
NKX3-2	18.77928025	3.614851073	0.331366282
GATA1	11.66764364	-1.734737093	0.159290057
NR2E1	14.90907715	4.655238649	0.427566876
EGR1	13244.96192	-1.866329649	0.171566784
ZNF724	72.65429307	1.8724693	0.172186943
FOXH1	12.88528376	3.2428993	0.29837428
CDX2	44.51248042	5.480191168	0.504741137
LHX5	18.47963767	5.239126365	0.483165765
MYCN	165.6222781	3.29569193	0.304565012
KLF17	8.356285491	-1.934646324	0.179093125
RFX8	35.40228573	2.160970116	0.200690945
IRF7	2372.775785	1.272921668	0.118493586
PRDM12	6.213075634	3.15914343	0.294265375
POU6F2	211.0500121	3.981410983	0.372415852
ZNF280B	168.76537	1.931022302	0.181395468
ZNF365	130.9937044	-1.850611227	0.174107575
ZIC5	26.2443618	5.591339367	0.526063016
DNTTIP1	3065.96818	1.534542068	0.1445076
GATA6	856.3147455	-1.74192037	0.164319848
HOXC12	53.12476749	7.869356711	0.742787399
HSF4	788.6209134	1.926031	0.181985498
FOXD2	113.6550809	1.277347716	0.121121811
ETS1	5785.531134	-1.177588064	0.112642287
NR4A3	942.8051648	-2.245831109	0.215489889
LHX1	17.12706062	5.700867474	0.547084335
PAX6	85.27325632	-1.332350886	0.128843409
E2F1	1007.404146	1.461566742	0.141900682
HLX	489.5390389	-1.263165478	0.123300681
HOXB7	493.0122256	2.084176521	0.204475197

ZBTB12	301.904561	1.214640583	0.119265343
FOXD3	13.86291136	4.121017299	0.404958471
NFE2L3	2121.804378	1.392500679	0.137234776
ZNF331	1346.597242	-1.192918464	0.118335923
ETV5	2669.270359	-1.168459607	0.115933722
GRHL1	1101.389973	1.555352772	0.154413249
TBX10	9.442832016	4.407557838	0.437624621
SOX5	139.6173284	-1.662722683	0.165540133
TWIST1	126.8828243	2.456358037	0.244562547
MITF	790.5137807	-1.167725	0.116701528
ASCL1	967.6447955	4.87610873	0.487942091
HOXA4	56.63192868	-1.728260913	0.17320527
HOXA13	22.91937755	5.803747701	0.582730706
ZEB2	2668.636383	-1.310490442	0.13191331
NFATC4	1750.089582	1.200007309	0.120799232
ATF3	3246.296656	-1.765126676	0.178214273
PRRX2	247.5413994	2.185715267	0.221082752
LIN28B	20.63044107	7.971077452	0.806642844
NFIX	4357.836188	-1.44292863	0.146369562
LHX6	172.8284288	-1.423636203	0.14467314
EGR3	520.2946016	-1.74656028	0.177556469
TIGD3	48.79160846	1.472758371	0.150857724
MIXL1	43.4338994	1.997406569	0.20562108
ZNF367	287.4793545	1.213002239	0.125523266
JUNB	9456.735125	-1.166868691	0.120759124
ZNF726	175.3782495	1.573302537	0.163065321
PRDM5	287.3207238	-1.155769856	0.119869003
PGR	163.7774024	-1.698741904	0.176481453
GCM1	15.61295306	2.704843736	0.281261847
DLX5	54.62196178	2.453525622	0.255702286
SP5	253.6194804	2.327786178	0.243333483
LCOR	1517.112177	1.072399575	0.112119026
ZMAT4	47.67430088	4.316014456	0.451856353
HOXC6	129.8073317	2.411059806	0.253432118
TEF	1534.187572	-1.157952364	0.12218935
NR0B1	194.0590234	6.078449856	0.646624255
FOS	18600.76935	-1.667595069	0.177925555
CDX1	17.34700053	1.809850233	0.19415449
FOXA1	3619.437715	1.39305056	0.149638135
HOXB8	128.4508339	3.345179118	0.361182212
DLX3	176.8672946	2.181394783	0.236010758
ZNF93	252.2896624	1.219308435	0.132574548
TET1	164.3667032	1.597309007	0.174697341
PPARG	1006.747331	-1.581005672	0.173074226
JUN	11124.92613	-1.092313628	0.119799521
HOXC8	45.12162749	2.729283596	0.299699254
ONECUT3	25.79530524	4.516293844	0.497266091
HEYL	1047.75524	-1.012952309	0.111960654
ZBTB8B	24.53625123	2.703661771	0.29928283
TLX1	6.611778869	4.617735252	0.514407635
SATB2	322.7134123	1.293287197	0.144155627
TFAP2C	951.1698533	1.215563302	0.136727947
ISL1	42.80095577	4.169764636	0.4691045

ZNF215	104.192697	1.497760397	0.169337784
TCF4	3057.000156	-1.103543489	0.12501734
HOXD4	20.9813167	2.865703664	0.324814005
CASZ1	941.8872507	-1.024555872	0.116689144
RXRG	91.03987503	-2.344889707	0.268567842
NR4A1	7061.388857	-1.760184454	0.201907973
SALL3	16.85593619	4.36703889	0.501129609
SCX	91.78209127	1.591558131	0.182645249
HOXA11	22.40011519	4.130031101	0.474677993
ZNF681	318.3927121	1.131129696	0.130015496
PRDM13	7.4633132	4.842729704	0.557899916
MEIS1	786.2977296	-1.067302135	0.123875568
TLX2	9.631295875	2.737619846	0.322385023
ZNF280A	9.225326434	5.001461216	0.589294429
DMRTC2	12.68136365	5.079554659	0.599968383
NME2	3009.154967	1.001063371	0.118948448
PLAGL1	880.0143991	-1.174094545	0.139975827
EMX1	10.9445012	2.986451248	0.356493379
SOX21	201.3607612	2.694716315	0.322160116
ALX3	9.163849139	4.838011099	0.584563933
NPAS2	1192.473584	1.227437139	0.14940933
HOXC4	108.3492026	1.683769393	0.205719743
ZNF117	2734.62112	1.386763785	0.169934149
GATA5	51.58131845	-1.569201487	0.192584559
HOXA1	86.94271778	2.034556724	0.250045906
DMRT1	5.691420639	3.962190214	0.492728714
MYBL1	359.1496389	1.196545168	0.149613149
EVX1	5.155011322	4.258069922	0.535077639
POU2AF1	1159.339213	1.574798979	0.197978173
HOXD13	15.30560444	5.822491631	0.736076256
TBX18	136.2626912	2.664185959	0.337476151
HOXD11	9.808499227	5.188375392	0.65799538
BATF	609.4012846	1.116516581	0.141711832
ZIC1	54.84495613	4.521663272	0.574048248
SMAD9	516.8445355	-1.410765774	0.179209047
ZBTB16	401.3109249	-2.097866368	0.266496994
FOXE3	5.223368591	3.108394888	0.395593807
FOXF2	392.8299823	-1.236869408	0.157414798
HEY2	116.3554741	-1.110775792	0.143491005
ZNF322	482.3579557	1.141740347	0.148012273
FOXL2	7.162601177	4.032754364	0.523156461
DLX1	5.785390302	2.821985645	0.366630965
ZNF572	135.0328208	1.059928964	0.137730514
NR5A2	216.3051451	-1.157313084	0.150581314
INSM1	48.98938009	2.975492331	0.387156987
TCF24	19.52921279	2.465303837	0.325426583
IRF8	1388.606114	-1.019289405	0.135252063
TBX6	157.0467424	1.123370238	0.149771577
HNF4A	306.8927071	2.883204901	0.384436402
ATOH7	6.777974373	2.203391999	0.294562952
ETV1	2213.147632	-1.168404351	0.156347801
ZNF648	6.315120394	3.008448682	0.403144346
SOX14	34.78114978	5.256558734	0.704738356

HELT	4.143366785	-3.156542025	0.425968082
LIN28A	8.525955164	4.10482791	0.555671223
SIM1	9.553249198	3.725437126	0.504850444
SOHLH1	4.700248447	4.024166709	0.545765712
NR5A1	6.427062298	2.815403069	0.383763245
SIM2	160.5312507	1.595604902	0.217615536
MYRF	2412.550026	-1.622202511	0.22194498
PAX5	255.8201113	1.697274091	0.235328176
ZIC4	16.95254443	4.757259956	0.660773395
ISL2	18.22782936	1.757166478	0.244120053
LHX8	7.007129286	4.348826099	0.606205946
HOXB3	900.9678275	1.390578096	0.193848536
MEF2B	28.93685135	1.135367273	0.158485105
DLX4	160.3537558	1.204305371	0.16848504
ZFHX4	178.9712459	1.765806866	0.247351312
NKX6-1	12.9183252	2.165618733	0.303396277
VAX2	49.21399237	1.459894353	0.2063205
ZNF732	21.67524597	1.937984414	0.273966408
KLF15	446.503868	-1.401998623	0.199114073
HES7	10.35903751	1.996509219	0.283982344
THRB	526.6233173	-1.076932882	0.153384143
GFI1B	10.07570317	-1.75176779	0.250278706
BHLHE41	1141.497695	-1.028977417	0.147170076
FOXG1	10.82455426	4.272096996	0.613361916
SP9	6.851235003	4.768643903	0.684979443
HOXB2	575.9478335	1.548245114	0.222457195
ZNF560	39.45762724	3.815392358	0.549982337
ZNF300	266.7359463	1.197853249	0.173014857
ZNF793	335.9146569	1.041492112	0.150690124
ZNF114	159.1852112	1.813422297	0.262435038
GSC	14.09313082	2.301531786	0.336470218
NR1I2	33.22887888	1.834029023	0.270007034
NEUROD1	40.63297217	4.735438706	0.698618183
ATOH8	1834.007364	-1.530639024	0.227048525
MSX2	103.1523975	1.386560306	0.205706062
OSR2	236.0121733	1.262315409	0.187310915
ALX1	14.76426274	3.14618378	0.468972674
ZNF730	40.50210277	1.785587662	0.267726085
PEG3	162.570359	-1.212069725	0.18280338
IRX4	6.843307195	3.880291812	0.585276762
SOX15	114.4438443	1.393824545	0.210693274
LHX4	89.09612908	1.038087461	0.157366762
EGR4	22.3536385	1.975328357	0.299588297
GBX2	8.488847063	2.625252823	0.39998099
VAX1	6.806807498	4.810732994	0.733725513
DLX2	3.937196307	2.925265605	0.447555528
TBR1	6.748824052	2.915099948	0.44660475
ZNF710	2482.842493	1.008474457	0.15493345
FOXD1	56.29057843	2.143222022	0.33009468
ZNF229	274.9527183	1.361490745	0.209797364
NPAS3	157.2597094	1.89756781	0.292730225
MYT1	56.76328552	2.044339393	0.316067119
MEOX2	274.1940616	-1.11057432	0.173159917

ZNF714	546.0120661	1.020804742	0.159630825
OVOL3	6.910376416	1.392554391	0.217907511
ZNF705A	2.001770067	-1.474489601	0.231020834
ZNF385B	915.3042138	-1.689000902	0.265507479
SOX30	38.67590512	1.564053834	0.246407957
PHOX2B	6.561516417	4.660823044	0.736646197
SOX9	1665.428119	1.369886191	0.217908366
SPIB	244.3905232	1.422170473	0.22707503
AIRE	4.747329986	1.716465932	0.274553878
ZNF716	7.084435534	4.412509779	0.711843626
LMX1B	65.77537138	1.944770541	0.314185589
RFX6	13.0332861	3.500814405	0.568369974
MSX1	78.67953611	-1.011553849	0.164995974
HLF	1187.930638	-1.363380119	0.222539034
TWIST2	125.630329	-1.0257554	0.16758324
ZNF486	1066.435499	1.355449642	0.223005936
ISX	11.90406907	3.740838392	0.619408997
ZNF474	62.25065663	-1.380448073	0.22894744
NOTO	14.9397313	2.65323977	0.440901834
DRGX	106.9621866	2.171474305	0.360877101
NKX2-4	4.50011382	3.959080977	0.659508687
FOXQ1	1000.074199	1.42235399	0.237755494
POU1F1	3.472320636	-1.240581047	0.207757065
RAX	2.995214639	3.268639733	0.550483276
ASCL2	147.3985459	1.278386685	0.215654609
DPF1	33.55791112	1.160236131	0.196703625
NKX2-5	4.656152067	3.265895226	0.558097653
OTX2	5.422349987	4.429745524	0.760514096
ZNF536	9.375701789	-1.745895905	0.304470719
SOX2	972.6886225	1.593397476	0.279137882
HMX2	3.014547851	3.38762303	0.59573261
CPEB1	58.33969116	-1.100086926	0.194293085
ZNF556	10.65884336	1.668662036	0.304593556
MEOX1	67.04026189	-1.167816115	0.214272551
HOXD1	198.7355582	1.441203527	0.265556051
POU5F1	176.8977895	1.257169721	0.233053229
ZIC3	4.894305427	4.087362873	0.761040795
ASCL5	45.59643266	1.115521905	0.208395468
HOXC5	5.570635487	1.644159723	0.309480978
ST18	36.11048836	1.26138831	0.237880548
HOXD12	1.848776645	3.080510053	0.581162354
GBX1	4.712079729	2.58850042	0.491708407
CCDC169-SOHLH2	1.652191051	2.145407737	0.408290152
CCDC17	332.0881438	-1.292507809	0.246903684
ARX	175.7280924	1.613515035	0.314319998
DMRT3	20.8948288	1.888554033	0.369267731
RBPJL	3.273085269	1.703028087	0.333175615
ZNF80	11.77921775	1.162223613	0.228635687
PHOX2A	2.671071864	3.105658257	0.615112231
PRDM9	3.141413049	3.792754346	0.751564945
ARID3C	4.823342229	1.291792993	0.256934638
ZNF750	658.846332	1.078222307	0.215374935
NKX6-3	3.54060133	1.899606155	0.393764945

SOX3	3.181306988	2.909906219	0.609820848
ZNF492	22.12707218	1.373843702	0.294966211
POU4F3	3.335384946	1.367314281	0.294359273
ZNF257	94.28348198	1.043447517	0.228167649
LTF	5565.79684	1.280934226	0.280761948
SOX1	5.940850587	3.282551212	0.732389692
PAX3	3.528556694	2.336850415	0.52622629
SP7	3.315209746	1.614928653	0.36576487
LHX3	1.300175515	2.257239317	0.525317829
ATOH1	2.562996904	3.500263093	0.821380924
NKX2-2	2.680099766	2.959454514	0.70097948
SCRT1	3.855532318	1.032908589	0.245967533
HOXD10	35.80577113	1.434520573	0.342051838
EN1	2.940193462	1.687136218	0.407304817
ZNF729	2.858305441	3.249210579	0.786002591
FOXN4	16.12258983	1.602391664	0.389692239
IRX1	129.8007593	-1.372667741	0.334899916
POU5F2	5.82946241	1.343339189	0.330842303
SKOR2	2.175325791	3.211467227	0.79580355
NEUROG2	1.224218161	2.221642823	0.551518884
SIX3	33.36739906	1.567832767	0.38938648
GATA4	20.40837692	1.80550972	0.449210986
NKX2-3	9.301396076	1.472803317	0.367532229
EVX2	1.374415255	2.592913239	0.653661262
ZNF488	35.30537415	1.155261639	0.293346143
ZNF676	35.72838963	1.280616039	0.328452824
TP63	703.5405758	1.041943293	0.271953306
ZNF99	8.878504657	1.30392826	0.347439357
SIX6	1.439831587	2.058830132	0.551010249
TBX20	4.467976318	1.793338452	0.482738138
VSX2	1.545177219	-1.147380601	0.324476674
BARHL2	1.360971796	2.487085819	0.711054343
CTCFL	7.095023992	1.545970408	0.442461242
ALX4	9.954957978	1.13168071	0.326322684
POU3F3	3.346115954	1.930177767	0.559896182
HOXB1	3.368151267	1.642082409	0.479423741
ZNF98	11.90361679	1.358987367	0.405377503
ZSCAN4	20.49252352	1.090923253	0.325907897
OLIG2	2.105075428	1.109793571	0.341261664
OTP	2.14387124	1.265905749	0.389852124
TLX3	1.714990361	2.451930822	0.760037912
TGIF2LX	2.128310671	3.335178905	1.03673039
ZFP42	77.41731736	1.583690484	0.492953973
OLIG3	2.736467297	3.340808568	1.044436918
TBPL2	0.92882011	1.325271132	0.427270959
NR1H4	23.47840359	1.186140689	0.415476541
PAX1	4.923640881	1.294504763	0.454156113
GSX2	0.9036835	1.406614262	0.494383739
NEUROG3	4.502036622	1.055685835	0.390464712
FOXR1	0.758120494	1.03079205	0.423571045
ESX1	1.113691111	2.28600181	0.982700024
TFAP2B	13.39249544	1.055834213	0.472394954
ZNF679	1.071891348	2.139268909	0.970569644

TFDP3	1.063475895	1.189979072	0.557819676

stat	pvalue	padj
-24.70709247	8.97E-135	4.05E-131
24.28736072	2.67E-130	8.03E-127
21.27780843	1.82E-100	5.03E-98
20.8123877	3.34E-96	6.94E-94
-20.30798412	1.09E-91	1.90E-89
-20.18126957	1.43E-90	2.35E-88
19.89761515	4.27E-88	5.98E-86
19.65297854	5.45E-86	6.66E-84
19.27688783	8.40E-83	8.77E-81
-19.16128199	7.79E-82	7.74E-80
-19.10648106	2.23E-81	2.18E-79
18.88345882	1.56E-79	1.38E-77
18.58713801	4.08E-77	3.34E-75
-18.44328727	5.90E-76	4.54E-74
18.38446052	1.75E-75	1.32E-73
17.84437908	3.20E-71	2.09E-69
-17.75391821	1.61E-70	1.03E-68
17.54116874	6.95E-69	4.20E-67
17.5316945	8.21E-69	4.94E-67
17.3882376	1.01E-67	5.72E-66
17.35251153	1.89E-67	1.05E-65
17.31886707	3.39E-67	1.87E-65
17.18653087	3.35E-66	1.79E-64
17.08907506	1.79E-65	9.35E-64
17.05907987	2.99E-65	1.54E-63
-16.64729123	3.17E-62	1.42E-60
16.21229879	4.13E-59	1.64E-57
-16.11600134	1.97E-58	7.60E-57
-16.11419701	2.03E-58	7.81E-57
16.04612769	6.08E-58	2.31E-56
16.02782288	8.17E-58	3.08E-56
-15.96579797	2.21E-57	8.21E-56
15.89767961	6.58E-57	2.41E-55
15.89600635	6.75E-57	2.47E-55
15.85636716	1.27E-56	4.59E-55
15.84688015	1.48E-56	5.32E-55
15.73514711	8.69E-56	3.01E-54
15.67602471	2.21E-55	7.57E-54
-15.27472926	1.13E-52	3.41E-51
15.22977118	2.24E-52	6.72E-51
15.18711009	4.30E-52	1.28E-50
15.12546785	1.10E-51	3.23E-50
15.11463015	1.30E-51	3.80E-50
-15.0633576	2.82E-51	8.20E-50
15.05386042	3.26E-51	9.42E-50
14.97577023	1.06E-50	2.99E-49
-14.90670806	2.98E-50	8.21E-49
14.76956227	2.30E-49	6.07E-48
14.75232667	2.97E-49	7.81E-48
14.55820844	5.18E-48	1.28E-46
14.45585717	2.30E-47	5.50E-46

-14.3233132	1.56E-46	3.61E-45
-14.30693642	1.98E-46	4.54E-45
14.25835485	3.98E-46	9.03E-45
14.2415208	5.06E-46	1.14E-44
-14.19622561	9.67E-46	2.16E-44
14.06942711	5.85E-45	1.25E-43
13.92595758	4.41E-44	9.00E-43
-13.74053379	5.80E-43	1.12E-41
13.72772993	6.93E-43	1.33E-41
13.66401464	1.67E-42	3.14E-41
-13.64495826	2.16E-42	4.06E-41
13.6371194	2.41E-42	4.52E-41
-13.61502849	3.26E-42	6.09E-41
13.61465832	3.28E-42	6.12E-41
-13.43427882	3.81E-41	6.75E-40
13.43059563	4.00E-41	7.08E-40
13.39849123	6.17E-41	1.08E-39
13.35703814	1.08E-40	1.86E-39
-13.32921279	1.57E-40	2.68E-39
13.31644738	1.86E-40	3.17E-39
13.2949767	2.48E-40	4.19E-39
13.29327305	2.53E-40	4.28E-39
13.28860723	2.70E-40	4.55E-39
-13.14589791	1.80E-39	2.89E-38
-13.12342057	2.42E-39	3.87E-38
-13.03052596	8.20E-39	1.28E-37
-13.02221046	9.15E-39	1.42E-37
13.01108934	1.06E-38	1.63E-37
12.96387987	1.96E-38	3.00E-37
12.91544276	3.68E-38	5.56E-37
12.88874908	5.21E-38	7.82E-37
-12.87972121	5.85E-38	8.76E-37
12.87523731	6.20E-38	9.27E-37
12.85641576	7.92E-38	1.18E-36
12.78664821	1.95E-37	2.85E-36
12.78504328	1.99E-37	2.90E-36
12.7673565	2.49E-37	3.62E-36
-12.74204303	3.45E-37	4.99E-36
12.69245367	6.51E-37	9.32E-36
12.65652912	1.03E-36	1.46E-35
-12.65428144	1.06E-36	1.50E-35
-12.63828396	1.30E-36	1.82E-35
-12.59833004	2.16E-36	3.00E-35
12.57829557	2.78E-36	3.84E-35
12.56194432	3.42E-36	4.69E-35
12.37375621	3.62E-35	4.76E-34
12.34736351	5.03E-35	6.56E-34
12.34393115	5.25E-35	6.82E-34
12.3438258	5.26E-35	6.83E-34
12.33368831	5.97E-35	7.70E-34
-12.18711628	3.64E-34	4.50E-33
12.17119753	4.43E-34	5.45E-33
11.94329875	7.04E-33	8.21E-32

-11.87428341	1.61E-32	1.84E-31
11.82783031	2.80E-32	3.17E-31
11.81852518	3.13E-32	3.53E-31
11.77313782	5.37E-32	5.96E-31
-11.74251813	7.72E-32	8.49E-31
11.67214309	1.77E-31	1.92E-30
-11.6351639	2.73E-31	2.93E-30
11.55030004	7.36E-31	7.72E-30
-11.50814111	1.20E-30	1.24E-29
-11.49818533	1.35E-30	1.39E-29
11.36641624	6.15E-30	6.13E-29
11.34560306	7.80E-30	7.74E-29
11.34479281	7.87E-30	7.81E-29
11.34081134	8.24E-30	8.16E-29
11.29112478	1.45E-29	1.42E-28
11.27816848	1.68E-29	1.64E-28
11.26916053	1.86E-29	1.81E-28
-11.22670296	3.02E-29	2.88E-28
11.11093356	1.11E-28	1.03E-27
-11.09766265	1.29E-28	1.19E-27
-11.08958322	1.41E-28	1.30E-27
11.03069701	2.72E-28	2.47E-27
11.00430276	3.64E-28	3.28E-27
10.91630237	9.63E-28	8.50E-27
10.90892848	1.04E-27	9.20E-27
-10.89042921	1.28E-27	1.12E-26
10.88774391	1.32E-27	1.15E-26
-10.87815255	1.47E-27	1.27E-26
10.87463002	1.52E-27	1.32E-26
10.86856179	1.63E-27	1.41E-26
10.85742923	1.84E-27	1.59E-26
10.84333111	2.15E-27	1.85E-26
10.82098008	2.74E-27	2.35E-26
-10.80246	3.35E-27	2.86E-26
10.76765132	4.89E-27	4.14E-26
10.74253645	6.43E-27	5.40E-26
10.73569538	6.92E-27	5.81E-26
10.69076669	1.12E-26	9.32E-26
10.64537239	1.83E-26	1.50E-25
-10.62912527	2.18E-26	1.78E-25
10.62864942	2.19E-26	1.78E-25
10.6191098	2.43E-26	1.97E-25
-10.60079101	2.95E-26	2.39E-25
10.59435946	3.17E-26	2.56E-25
10.58343123	3.56E-26	2.86E-25
10.54597601	5.30E-26	4.23E-25
-10.45422724	1.40E-25	1.09E-24
-10.42197904	1.97E-25	1.53E-24
10.4204546	2.00E-25	1.55E-24
-10.34085404	4.60E-25	3.51E-24
10.29992754	7.05E-25	5.33E-24
-10.24459452	1.25E-24	9.31E-24
10.19280849	2.14E-24	1.56E-23

10.18435492	2.33E-24	1.70E-23
10.17639486	2.53E-24	1.85E-23
10.14684992	3.42E-24	2.49E-23
-10.08078053	6.72E-24	4.79E-23
-10.07868619	6.86E-24	4.89E-23
10.07266398	7.30E-24	5.19E-23
10.07154905	7.38E-24	5.24E-23
-10.04422708	9.74E-24	6.87E-23
10.04388474	9.77E-24	6.89E-23
-10.00608148	1.43E-23	1.00E-22
9.99321195	1.63E-23	1.14E-22
-9.978108128	1.90E-23	1.32E-22
9.959570758	2.29E-23	1.58E-22
-9.934482321	2.95E-23	2.03E-22
9.933898503	2.96E-23	2.04E-22
-9.904519148	3.98E-23	2.72E-22
9.88641241	4.77E-23	3.24E-22
9.881792806	4.99E-23	3.39E-22
-9.858119457	6.32E-23	4.27E-22
-9.840362913	7.54E-23	5.06E-22
-9.836646826	7.83E-23	5.25E-22
9.762565217	1.63E-22	1.07E-21
9.714016517	2.63E-22	1.70E-21
9.663564952	4.31E-22	2.74E-21
-9.662778699	4.34E-22	2.76E-21
9.648296324	5.00E-22	3.17E-21
-9.641941027	5.32E-22	3.37E-21
-9.625611519	6.23E-22	3.93E-21
9.616817073	6.79E-22	4.27E-21
9.595243212	8.37E-22	5.24E-21
9.566238684	1.11E-21	6.90E-21
9.564831371	1.12E-21	6.99E-21
9.551740132	1.28E-21	7.90E-21
9.513631579	1.84E-21	1.13E-20
-9.476704477	2.62E-21	1.59E-20
9.400281245	5.44E-21	3.23E-20
-9.372431446	7.09E-21	4.18E-20
9.321701656	1.14E-20	6.67E-20
9.309462188	1.28E-20	7.47E-20
9.261749354	2.01E-20	1.15E-19
9.242776899	2.40E-20	1.37E-19
9.197153272	3.68E-20	2.07E-19
9.143293185	6.06E-20	3.37E-19
-9.134841768	6.55E-20	3.63E-19
-9.117846369	7.66E-20	4.23E-19
9.10674138	8.49E-20	4.67E-19
9.082247766	1.06E-19	5.82E-19
-9.047395442	1.46E-19	7.97E-19
9.03380181	1.66E-19	8.98E-19
8.976801548	2.79E-19	1.49E-18
8.971465258	2.93E-19	1.56E-18
8.890379249	6.09E-19	3.18E-18
8.888775603	6.18E-19	3.22E-18

8.844809264	9.17E-19	4.72E-18
-8.827123443	1.07E-18	5.51E-18
8.822598839	1.12E-18	5.73E-18
-8.780215864	1.63E-18	8.29E-18
-8.731088918	2.52E-18	1.27E-17
-8.717756035	2.84E-18	1.43E-17
8.714390076	2.92E-18	1.47E-17
8.713931173	2.94E-18	1.47E-17
8.700700605	3.30E-18	1.65E-17
8.699960599	3.32E-18	1.66E-17
8.68028399	3.95E-18	1.96E-17
-8.615921203	6.94E-18	3.40E-17
8.491771176	2.04E-17	9.66E-17
8.487202612	2.12E-17	1.00E-16
8.466370572	2.53E-17	1.19E-16
8.415943097	3.90E-17	1.81E-16
-8.387837885	4.95E-17	2.28E-16
8.377297936	5.42E-17	2.49E-16
8.364524906	6.04E-17	2.77E-16
8.276273693	1.27E-16	5.73E-16
8.215264318	2.12E-16	9.41E-16
8.184772966	2.73E-16	1.20E-15
8.160595125	3.33E-16	1.47E-15
-8.148116846	3.70E-16	1.62E-15
8.136732782	4.06E-16	1.77E-15
8.041321935	8.89E-16	3.80E-15
7.99759365	1.27E-15	5.37E-15
7.957854364	1.75E-15	7.35E-15
7.954407059	1.80E-15	7.54E-15
7.910174505	2.57E-15	1.07E-14
7.89444218	2.92E-15	1.20E-14
7.88512435	3.14E-15	1.29E-14
7.878781618	3.31E-15	1.36E-14
7.876800052	3.36E-15	1.38E-14
-7.872179453	3.49E-15	1.43E-14
-7.872007616	3.49E-15	1.43E-14
7.857541835	3.92E-15	1.60E-14
-7.857389662	3.92E-15	1.60E-14
-7.74108311	9.86E-15	3.93E-14
7.713822106	1.22E-14	4.83E-14
7.708505319	1.27E-14	5.02E-14
7.697073931	1.39E-14	5.47E-14
7.695672776	1.41E-14	5.53E-14
-7.685635463	1.52E-14	5.96E-14
7.685493038	1.52E-14	5.96E-14
7.575606806	3.57E-14	1.37E-13
-7.536220759	4.84E-14	1.83E-13
7.50055694	6.35E-14	2.39E-13
7.499822828	6.39E-14	2.40E-13
7.480207499	7.42E-14	2.78E-13
-7.473110206	7.83E-14	2.93E-13
7.462460311	8.49E-14	3.17E-13
7.458879867	8.73E-14	3.25E-13

-7.410278282	1.26E-13	4.66E-13
7.387152229	1.50E-13	5.52E-13
7.379288596	1.59E-13	5.84E-13
7.373432631	1.66E-13	6.09E-13
7.33630202	2.20E-13	7.97E-13
7.332219608	2.26E-13	8.21E-13
-7.309029978	2.69E-13	9.72E-13
7.2123709	5.50E-13	1.95E-12
7.199533139	6.04E-13	2.14E-12
7.19796041	6.11E-13	2.16E-12
7.173842698	7.29E-13	2.56E-12
7.173529019	7.31E-13	2.56E-12
7.163873659	7.84E-13	2.74E-12
7.1478475	8.81E-13	3.08E-12
7.138861928	9.41E-13	3.28E-12
7.137921251	9.48E-13	3.30E-12
7.075856989	1.49E-12	5.09E-12
7.073803067	1.51E-12	5.16E-12
-7.041182985	1.91E-12	6.49E-12
7.030399118	2.06E-12	7.00E-12
-7.021148725	2.20E-12	7.46E-12
-6.999268199	2.57E-12	8.67E-12
-6.991757037	2.71E-12	9.14E-12
6.96505095	3.28E-12	1.10E-11
6.961732874	3.36E-12	1.12E-11
6.959743929	3.41E-12	1.14E-11
6.937299804	4.00E-12	1.33E-11
6.923412651	4.41E-12	1.46E-11
6.911482218	4.80E-12	1.58E-11
6.909985474	4.85E-12	1.60E-11
6.840224379	7.91E-12	2.58E-11
6.792523132	1.10E-11	3.55E-11
6.778292947	1.22E-11	3.90E-11
-6.741462095	1.57E-11	4.99E-11
6.740493171	1.58E-11	5.02E-11
6.739144963	1.59E-11	5.06E-11
6.708671854	1.96E-11	6.20E-11
6.669457184	2.57E-11	8.02E-11
-6.630455751	3.35E-11	1.04E-10
6.629840901	3.36E-11	1.04E-10
6.615420216	3.70E-11	1.14E-10
6.596611946	4.21E-11	1.29E-10
6.593476373	4.30E-11	1.32E-10
6.563443981	5.26E-11	1.60E-10
6.556584046	5.51E-11	1.68E-10
6.53609535	6.31E-11	1.91E-10
6.527247964	6.70E-11	2.03E-10
6.509081516	7.56E-11	2.28E-10
6.492749356	8.43E-11	2.53E-10
6.489551238	8.61E-11	2.58E-10
6.482309135	9.03E-11	2.70E-10
6.46805463	9.93E-11	2.96E-10
-6.413576202	1.42E-10	4.19E-10

6.394784611	1.61E-10	4.72E-10
6.390575446	1.65E-10	4.85E-10
-6.382496219	1.74E-10	5.10E-10
-6.361406118	2.00E-10	5.83E-10
6.347416098	2.19E-10	6.36E-10
6.327084922	2.50E-10	7.22E-10
6.286524073	3.25E-10	9.32E-10
6.26299807	3.78E-10	1.08E-09
6.251836416	4.06E-10	1.15E-09
6.198706596	5.69E-10	1.60E-09
6.189878248	6.02E-10	1.69E-09
6.159393646	7.30E-10	2.04E-09
-6.13077899	8.74E-10	2.42E-09
-6.126476309	8.98E-10	2.49E-09
-6.120871047	9.31E-10	2.57E-09
6.078087722	1.22E-09	3.33E-09
6.039367219	1.55E-09	4.21E-09
-6.029541418	1.64E-09	4.46E-09
6.017756251	1.77E-09	4.79E-09
6.017212778	1.77E-09	4.81E-09
6.00307631	1.94E-09	5.23E-09
5.982423239	2.20E-09	5.90E-09
-5.971306179	2.35E-09	6.30E-09
5.937763914	2.89E-09	7.68E-09
5.927935833	3.07E-09	8.14E-09
5.898397295	3.67E-09	9.67E-09
5.851834728	4.86E-09	1.27E-08
5.824672479	5.72E-09	1.48E-08
-5.734199701	9.80E-09	2.49E-08
5.708281038	1.14E-08	2.88E-08
5.686482443	1.30E-08	3.26E-08
-5.661997312	1.50E-08	3.74E-08
5.478323499	4.29E-08	1.04E-07
-5.450143326	5.03E-08	1.21E-07
5.427116122	5.73E-08	1.37E-07
5.394345865	6.88E-08	1.64E-07
5.370753973	7.84E-08	1.86E-07
5.352908666	8.66E-08	2.04E-07
5.312635806	1.08E-07	2.52E-07
5.302612253	1.14E-07	2.66E-07
5.300601511	1.15E-07	2.69E-07
5.264299704	1.41E-07	3.25E-07
5.254615442	1.48E-07	3.42E-07
-5.234866438	1.65E-07	3.79E-07
5.133351499	2.85E-07	6.38E-07
5.114321869	3.15E-07	7.03E-07
5.111502792	3.20E-07	7.13E-07
5.083299231	3.71E-07	8.23E-07
5.048929444	4.44E-07	9.79E-07
5.046475853	4.50E-07	9.91E-07
5.027710567	4.96E-07	1.09E-06
5.006257135	5.55E-07	1.21E-06
4.824213483	1.41E-06	2.97E-06

4.771739483	1.83E-06	3.83E-06
4.657630772	3.20E-06	6.56E-06
4.645052509	3.40E-06	6.95E-06
4.573161533	4.80E-06	9.69E-06
4.562349822	5.06E-06	1.02E-05
4.481973529	7.40E-06	1.47E-05
4.440770935	8.96E-06	1.77E-05
4.415209838	1.01E-05	1.98E-05
4.296902168	1.73E-05	3.33E-05
4.261437039	2.03E-05	3.89E-05
4.221884658	2.42E-05	4.60E-05
4.199369628	2.68E-05	5.06E-05
4.193868922	2.74E-05	5.18E-05
4.142195594	3.44E-05	6.43E-05
4.133842069	3.57E-05	6.66E-05
4.111941431	3.92E-05	7.30E-05
-4.098740175	4.15E-05	7.71E-05
4.060361009	4.90E-05	9.03E-05
4.035502516	5.45E-05	0.000100011
4.02822621	5.62E-05	0.000103
4.02641809	5.66E-05	0.000103752
4.019291109	5.84E-05	0.000106833
4.007276647	6.14E-05	0.000112005
3.966753717	7.29E-05	0.000131873
3.938219977	8.21E-05	0.000147707
3.898934481	9.66E-05	0.000172543
3.831331588	0.000127452	0.000224722
3.752966481	0.000174754	0.000303857
3.736464312	0.000186626	0.000323472
3.714930127	0.00020326	0.000351057
-3.536095788	0.000406087	0.000679042
3.49774366	0.000469212	0.000778044
3.49402447	0.000475797	0.000788318
3.467980512	0.000524385	0.000865561
3.447385118	0.000566041	0.000931342
3.425117004	0.000614535	0.001007828
3.352399571	0.000801143	0.001299242
3.34733605	0.000815922	0.001321668
3.252031173	0.001145835	0.00182612
3.247143393	0.001165696	0.001855156
3.226063838	0.001255054	0.001988782
3.21701663	0.001295311	0.002048802
3.212653859	0.001315147	0.002078358
3.198669552	0.001380633	0.002175948
3.101711231	0.001924055	0.002986823
2.854892084	0.004305145	0.006397342
2.85035195	0.004367088	0.006485671
2.84518715	0.004438534	0.006584742
2.703665152	0.006857936	0.009929474
2.433575344	0.014950524	0.020822421
2.326245805	0.020005441	0.027400207
2.235066662	0.025412973	0.034322708
2.20413746	0.027514678	0.037000875

2.133268374	0.032902717	0.043793548
