# Supplementary material for: Integrative Molecular Analyses of an Individual Transcription Factor-Based Genomic Model for Lung Cancer Prognosis
Source: Dis Markers. 2021 Dec 7;2021:5125643. doi: 10.1155/2021/5125643 (PMC8672105; doi:10.1155/2021/5125643)
Supplement: Supplementary 4 — Supplementary Table 4: determination of downstream targets of TFs: SATB2, HLF, and NPAS2. [file 5125643.f4.pdf]

Supplementary table 4. Determination of downstream targets of TFs: SATB2, HLF and NPAS2.

TF	Gene	CHEA	ENCODE	JASPAR	MotifMap
HLF	NEUROD6	0	0	0	0
HLF	PIK3R1	0	0	0	0
HLF	TNF	0	0	1	0
HLF	ENSA	0	0	0	0
HLF	ZNF687	0	0	0	0
HLF	AKTIP	0	0	0	0
HLF	NFKBIZ	0	0	0	0
HLF	MAP4	0	0	0	0
HLF	ZDHHC14	0	0	0	0
HLF	BEST3	0	0	0	0
HLF	ALB	0	0	0	0
HLF	MIR3648-1	0	0	1	0
HLF	NBPF14	0	0	1	0
HLF	BHLHE40	0	0	0	0
HLF	TRIM26	0	0	1	0
HLF	NYX	0	0	0	0
HLF	DMD	0	0	0	0
HLF	HCG26	0	0	1	0
HLF	DCAF6	0	0	0	0
HLF	TNNC2	0	0	0	0
HLF	IRF2BP1	0	0	0	0
HLF	IP6K2	0	0	0	0
HLF	RBFOX2	0	0	0	0
HLF	EPB41	0	0	0	0
HLF	GTPBP6	0	0	1	0
HLF	FBXO3	0	0	0	0
HLF	AQP9	0	0	0	0
HLF	CSNK1E	0	0	0	0
HLF	LONRF3	0	0	0	0
HLF	LOX	0	0	0	0
HLF	LGSN	0	0	0	0
HLF	ZFP57	0	0	1	0
HLF	CDKN2C	0	0	0	0
HLF	EPN2	0	0	0	0
HLF	LIPI	0	0	1	0
HLF	UBE2E2	0	0	0	0
HLF	HERPUD1	0	0	0	0
HLF	ABHD11	0	0	0	0
HLF	DDX39B	0	0	1	0
HLF	PDAP1	0	0	0	0
HLF	ATAD2	0	0	0	0
HLF	HOXA4	0	0	0	0
HLF	FOXA1	0	0	0	0
HLF	ANKRD40	0	0	0	0
HLF	ARRDC3	0	0	0	0
HLF	MECP2	0	0	0	0
HLF	SLC24A2	0	0	0	0
HLF	PNMA1	0	0	0	0
HLF	MEOX2	0	0	0	0
HLF	CD40LG	0	0	0	0
HLF	HS6ST2	0	0	0	0

HLF	HOXD9	0	0	1	0
HLF	REXO1L1P	0	0	1	0
HLF	CHST2	0	0	1	0
HLF	TENC1	0	0	0	0
HLF	PRKAA2	0	0	0	0
HLF	FCHSD2	0	0	0	0
HLF	HNRNPH1	0	0	0	0
HLF	SMARCAD1	0	0	0	0
HLF	FOXA2	0	0	0	0
HLF	SLIT3	0	0	0	0
HLF	CYP26A1	0	0	0	0
HLF	NR2F2	0	0	0	0
HLF	ZBTB18	0	0	0	0
HLF	PDGFRL	0	0	0	0
HLF	ACSL4	0	0	0	0
HLF	S100PBP	0	0	0	0
HLF	IL4	0	0	0	0
HLF	ABHD8	0	0	0	0
HLF	HOXB6	0	0	0	0
HLF	PHC3	0	0	0	0
HLF	NREP	0	0	0	0
HLF	YWHAG	0	0	0	0
HLF	RMDN3	0	0	0	0
HLF	VNN3	0	0	0	0
HLF	HNRNPA3	0	0	0	0
HLF	IFNG	0	0	0	0
HLF	CLCN5	0	0	0	0
HLF	PPL	0	0	0	0
HLF	NAA60	0	0	0	0
HLF	HIVEP3	0	0	0	0
HLF	OTP	0	0	0	0
HLF	TRIM40	0	0	1	0
HLF	TNXB	0	0	1	0
HLF	FOXP1	0	0	0	0
HLF	TFEB	0	0	0	0
HLF	ROBO1	0	0	0	0
HLF	TAGLN3	0	0	0	0
HLF	EGFR	0	0	0	0
HLF	SLC25A26	0	0	1	0
HLF	TREX1	0	0	0	0
HLF	ADNP	0	0	0	0
HLF	OR5T3	0	0	1	0
HLF	ZDHHC16	0	0	1	0
HLF	CHN1	0	0	0	0
HLF	DPH3	0	0	0	0
HLF	MPP6	0	0	0	0
HLF	CTDSP1	0	0	0	0
HLF	KRT23	0	0	0	0
HLF	RASGEF1A	0	0	0	0
HLF	TXLNG	0	0	0	0
HLF	ELMOD1	0	0	0	0
HLF	TMEM154	0	0	0	0
HLF	PLCB1	0	0	0	0

HLF	MIOX	0	0	0	0
HLF	ZBTB21	0	0	0	0
HLF	CELF2	0	0	0	0
HLF	FCGR1B	0	0	1	0
HLF	ETV4	0	0	0	0
HLF	GRHL2	0	0	0	0
HLF	TSC22D3	0	0	0	0
HLF	POGZ	0	0	0	0
HLF	ARHGAP44	0	0	0	0
HLF	ZNF711	0	0	0	0
HLF	CDH10	0	0	0	0
HLF	OXNAD1	0	0	0	0
HLF	FGF21	0	0	0	0
HLF	RHOBTB2	0	0	0	0
HLF	GTPBP2	0	0	0	0
HLF	CACNG2	0	0	0	0
HLF	PIP5K1A	0	0	0	0
HLF	EHMT2	0	0	1	0
HLF	SPIB	0	0	0	0
HLF	JPH3	0	0	0	0
HLF	ZDHHC5	0	0	0	0
HLF	PDP1	0	0	0	0
HLF	RPS6KA3	0	0	0	0
HLF	HOXC4	0	0	0	0
HLF	SPRR1B	0	0	0	0
HLF	HCP5	0	0	1	0
HLF	XPO7	0	0	0	0
HLF	NEUROD1	0	0	0	0
HLF	NTRK1	0	0	0	0
HLF	ALDH1A1	0	0	0	0
HLF	GPR112	0	0	0	0
HLF	EML4	0	0	0	0
HLF	SECISBP2L	0	0	0	0
HLF	NEU1	0	0	1	0
HLF	NPVF	0	0	0	0
HLF	ANO1	0	0	0	0
HLF	ANGPTL1	0	0	0	0
HLF	CTSC	0	0	0	0
HLF	CHD2	0	0	0	0
HLF	TNFSF13	0	0	0	0
HLF	RNF38	0	0	1	0
HLF	CDKL5	0	0	0	0
HLF	TTC39B	0	0	0	0
HLF	ZFYVE9	0	0	0	0
HLF	KLF9	0	0	0	0
HLF	CDC42	0	0	0	0
HLF	LRRTM4	0	0	0	0
HLF	FNBP1	0	0	0	0
HLF	IL27	0	0	0	0
HLF	CLIC4	0	0	0	0
HLF	INPP4A	0	0	0	0
HLF	ATP6V1G2-DDX39B	0	0	1	0
HLF	PCBP2	0	0	0	0

HLF	FAIM2	0	0	0	0
HLF	SHMT2	0	0	0	0
HLF	PFN2	0	0	0	0
HLF	LINC00518	0	0	1	0
HLF	LSM2	0	0	1	0
HLF	CADM1	0	0	0	0
HLF	ZDHHC2	0	0	0	0
HLF	BUD31	0	0	0	0
HLF	SPRED1	0	0	0	0
HLF	TSC22D1	0	0	0	0
HLF	KLHL7	0	0	0	0
HLF	NDST3	0	0	0	0
HLF	STK35	0	0	0	0
HLF	CALM1	0	0	0	0
HLF	CALCRL	0	0	0	0
HLF	DNAJA2	0	0	0	0
HLF	ELAVL4	0	0	0	0
HLF	PTHLH	0	0	0	0
HLF	WNT3A	0	0	0	0
HLF	RORA	0	0	0	0
HLF	CDK8	0	0	0	0
HLF	TMEM57	0	0	0	0
HLF	SREBF2	0	0	0	0
HLF	ARID1B	0	0	0	0
HLF	AP1S2	0	0	0	0
HLF	GBX2	0	0	0	0
HLF	LGALSL	0	0	0	0
HLF	FRS3	0	0	1	0
HLF	USP9X	0	0	0	0
HLF	LOC388942	0	0	1	0
HLF	FLOT1	0	0	1	0
HLF	BAMBI	0	0	0	0
HLF	NFIL3	0	0	0	0
HLF	PKNOX2	0	0	0	0
HLF	EYA1	0	0	1	0
HLF	SEMA6D	0	0	0	0
HLF	DDX5	0	0	0	0
HLF	OSBPL6	0	0	0	0
HLF	DLX1	0	0	0	0
HLF	SETD7	0	0	0	0
HLF	RUNX1	0	0	0	0
HLF	PRKG1	0	0	0	0
HLF	ZNF641	0	0	1	0
HLF	PRRC2A	0	0	1	0
HLF	CLASP1	0	0	0	0
HLF	SULF1	0	0	0	0
HLF	PLAG1	0	0	0	0
HLF	XK	0	0	0	0
HLF	HSPA4L	0	0	0	0
HLF	RARB	0	0	1	0
HLF	TCEAL7	0	0	0	0
HLF	PDE4D	0	0	0	0
HLF	TOP1	0	0	0	0

HLF	CREB5	0	0	0	0
HLF	RBFOX1	0	0	0	0
HLF	MYL6B	0	0	0	0
HLF	DLX5	0	0	0	0
HLF	HRH3	0	0	0	0
HLF	FAM21A	0	0	1	0
HLF	NUFIP2	0	0	0	0
HLF	SUMO1	0	0	0	0
HLF	DENND4A	0	0	0	0
HLF	TCF19	0	0	1	0
HLF	SSBP2	0	0	1	0
HLF	GPR89B	0	0	1	0
HLF	RPRD2	0	0	0	0
HLF	HIST3H2BB	0	0	0	0
HLF	PELI2	0	0	0	0
HLF	MIR30B	0	0	1	0
HLF	POU4F1	0	0	0	0
HLF	FBXW4	0	0	0	0
HLF	FOXO3	0	0	0	0
HLF	PDXP	0	0	0	0
HLF	CXCL6	0	0	0	0
HLF	SPRY4	0	0	0	0
HLF	BRD2	0	0	1	0
HLF	PARP4	0	0	1	0
HLF	PDZD8	0	0	0	0
HLF	MAP2K6	0	0	0	0
HLF	SH2B3	0	0	0	0
HLF	DBP	0	0	0	0
HLF	HOXC6	0	0	0	0
HLF	LINC00114	0	0	0	0
HLF	HIST3H2A	0	0	0	0
HLF	MPP2	0	0	0	0
HLF	RGR	0	0	0	0
HLF	BAI3	0	0	0	0
HLF	GCN1L1	0	0	1	0
HLF	PCSK2	0	0	0	0
HLF	MRC2	0	0	0	0
HLF	AGO3	0	0	0	0
HLF	DDIT3	0	0	0	0
HLF	C12ORF65	0	0	1	0
HLF	C9ORF24	0	0	0	0
HLF	USP2	0	0	0	0
HLF	PREX2	0	0	0	0
HLF	CCDC47	0	0	0	0
HLF	OCRL	0	0	0	0
HLF	CRIM1	0	0	0	0
HLF	CDYL	0	0	0	0
HLF	PRKAG1	0	0	0	0
HLF	CASS4	0	0	0	0
HLF	MILR1	0	0	1	0
HLF	LEMD2	0	0	0	0
HLF	CACNA2D3	0	0	0	0
HLF	ERF	0	0	0	0

HLF	F5	0	0	0	0
HLF	ING1	0	0	0	0
HLF	LINC00685	0	0	1	0
HLF	REXO1L2P	0	0	1	0
HLF	ZNF654	0	0	0	0
HLF	MIR3687-1	0	0	1	0
HLF	MBNL2	0	0	0	0
HLF	FAM101B	0	0	1	0
HLF	MEIS2	0	0	0	0
HLF	CDX2	0	0	1	0
HLF	CASK	0	0	0	0
HLF	BSN	0	0	0	0
HLF	TBX6	0	0	0	0
HLF	MKNK2	0	0	0	0
HLF	LOC401242	0	0	1	0
HLF	TOB1	0	0	0	0
HLF	CNTF	0	0	0	0
HLF	DUSP14	0	0	0	0
HLF	HLA-E	0	0	1	0
HLF	TNFSF15	0	0	0	0
HLF	ECM2	0	0	0	0
HLF	RCC2	0	0	0	0
HLF	ELMSAN1	0	0	0	0
HLF	SYT11	0	0	0	0
HLF	VARS	0	0	1	0
HLF	EIF4A1	0	0	0	0
HLF	ATXN1	0	0	0	0
HLF	EEF2	0	0	0	0
HLF	CYP21A2	0	0	1	0
HLF	NAPEPLD	0	0	0	0
HLF	TRIM8	0	0	0	0
HLF	SERTAD4	0	0	0	0
HLF	ZBTB12	0	0	0	0
HLF	ADRB2	0	0	0	0
HLF	OTX2	0	0	0	0
HLF	NR1D1	0	0	0	0
HLF	GNL1	0	0	1	0
HLF	TAC1	0	0	0	0
HLF	COL15A1	0	0	0	0
HLF	RNF39	0	0	1	0
HLF	BCL6	0	0	0	0
HLF	KRTAP19-5	0	0	0	0
HLF	NPTX1	0	0	0	0
HLF	RASL10B	0	0	0	0
NPAS2	CRY2	0	0	0	0
NPAS2	PER1	0	0	0	0
NPAS2	PER2	0	0	0	0
NPAS2	CRY1	0	0	0	0
SATB2	UPF3B	0	0	0	0
SATB2	TP63	0	0	0	0

TRANSFAC	TRRUST	Sum
1	0	1
1	0	1
0	0	1
1	0	1
1	0	1
1	0	1
1	0	1
1	0	1
1	0	1
1	0	1
1	0	1
0	0	1
0	0	1
1	0	1
0	0	1
1	0	1
1	0	1
0	0	1
1	0	1
1	0	1
1	0	1
1	0	1
1	0	1
1	0	1
0	0	1
1	0	1
1	0	1
1	0	1
1	0	1
1	0	1
1	0	1
0	0	1
1	0	1
1	0	1
0	0	1
1	0	1
1	0	1
1	0	1
0	0	1
1	0	1
1	0	1
1	0	1
1	0	1
1	0	1
1	0	1
1	0	1
1	0	1
1	0	1
1	0	1
1	0	1
1	0	1

0	0	1
0	0	1
0	0	1
1	0	1
1	0	1
1	0	1
1	0	1
1	0	1
1	0	1
1	0	1
1	0	1
1	0	1
1	0	1
1	0	1
1	0	1
1	0	1
1	0	1
1	0	1
1	0	1
1	0	1
1	0	1
1	0	1
1	0	1
1	0	1
1	0	1
1	0	1
1	0	1
1	0	1
1	0	1
1	0	1
1	0	1
0	0	1
0	0	1
1	0	1
1	0	1
1	0	1
1	0	1
1	0	1
0	0	1
1	0	1
1	0	1
0	0	1
0	0	1
1	0	1
1	0	1
1	0	1
1	0	1
1	0	1
1	0	1
1	0	1
1	0	1
1	0	1
1	0	1

1	0	1
1	0	1
1	0	1
0	0	1
1	0	1
1	0	1
1	0	1
1	0	1
1	0	1
1	0	1
1	0	1
1	0	1
1	0	1
1	0	1
1	0	1
1	0	1
1	0	1
0	0	1
1	0	1
1	0	1
1	0	1
1	0	1
1	0	1
1	0	1
1	0	1
0	0	1
1	0	1
1	0	1
1	0	1
1	0	1
1	0	1
1	0	1
1	0	1
0	0	1
1	0	1
1	0	1
1	0	1
1	0	1
1	0	1
1	0	1
0	0	1
1	0	1
1	0	1
1	0	1
1	0	1
1	0	1
1	0	1
1	0	1
1	0	1
1	0	1
1	0	1
0	0	1
1	0	1

1	0	1
1	0	1
1	0	1
0	0	1
0	0	1
1	0	1
1	0	1
1	0	1
1	0	1
1	0	1
1	0	1
1	0	1
1	0	1
1	0	1
1	0	1
1	0	1
1	0	1
1	0	1
1	0	1
1	0	1
1	0	1
1	0	1
1	0	1
1	0	1
1	0	1
1	0	1
1	0	1
0	0	1
1	0	1
0	0	1
0	0	1
1	0	1
1	0	1
1	0	1
0	0	1
1	0	1
1	0	1
1	0	1
1	0	1
1	0	1
0	1	1
1	0	1
0	0	1
0	0	1
1	0	1
1	0	1
1	0	1
1	0	1
1	0	1
0	0	1
1	0	1
1	0	1
1	0	1

1	0	1
1	0	1
1	0	1
1	0	1
1	0	1
0	0	1
1	0	1
1	0	1
1	0	1
0	0	1
0	0	1
0	0	1
1	0	1
1	0	1
1	0	1
0	0	1
1	0	1
1	0	1
1	0	1
1	0	1
1	0	1
1	0	1
0	0	1
0	0	1
1	0	1
1	0	1
1	0	1
0	1	1
1	0	1
1	0	1
1	0	1
1	0	1
1	0	1
1	0	1
0	0	1
1	0	1
1	0	1
1	0	1
1	0	1
0	0	1
1	0	1
1	0	1
1	0	1
1	0	1
1	0	1
1	0	1
1	0	1
1	0	1
1	0	1
0	0	1
1	0	1
1	0	1
1	0	1

1	0	1
1	0	1
0	0	1
0	0	1
1	0	1
0	0	1
1	0	1
0	0	1
1	0	1
0	0	1
1	0	1
1	0	1
1	0	1
1	0	1
0	0	1
1	0	1
1	0	1
1	0	1
0	0	1
1	0	1
1	0	1
1	0	1
1	0	1
1	0	1
0	0	1
1	0	1
1	0	1
1	0	1
0	0	1
1	0	1
1	0	1
1	0	1
1	0	1
1	0	1
1	0	1
1	0	1
0	0	1
1	0	1
1	0	1
0	0	1
1	0	1
1	0	1
1	0	1
1	0	1
0	1	1
0	1	1
0	1	1
0	1	1
0	1	1
0	1	1
